# Oxyethylated Fluoresceine—(thia)calix[4]arene Conjugates: Synthesis and Visible-Light Photoredox Catalysis in Water–Organic Media

**DOI:** 10.3390/molecules28010261

**Published:** 2022-12-28

**Authors:** Vladimir Burilov, Aigul Fatykhova, Diana Mironova, Elza Sultanova, Ramil Nugmanov, Alina Artemenko, Anastasia Volodina, Amina Daminova, Vladimir Evtugyn, Svetlana Solovieva, Igor Antipin

**Affiliations:** 1 Alexander Butlerov Institute of Chemistry, Kazan Federal University, 18 Kremlevskaya St., 420008 Kazan, Russia; 2 Interdisciplinary Center for Analytical Microscopy, Kazan Federal University, 18 Kremlevskaya St., 420008 Kazan, Russia; 3 Arbuzov Institute of Organic and Physical Chemistry, FRC Kazan Scientific Center of RAS, 8 Arbuzov Str., 420088 Kazan, Russia

**Keywords:** calixarene, thiacalixarene, fluorescein, luminescence, click chemistry, photoredox catalysis

## Abstract

Fluorescent derivatives attract the attention of researchers for their use as sensors, photocatalysts and for the creation of functional materials. In order to create amphiphilic fluorescent derivatives of calixarenes, a fluorescein derivative containing oligoethylene glycol and propargyl groups was obtained. The resulting fluorescein derivative was introduced into three different (thia)calix[4]arene azide derivatives. For all synthesized compounds, the luminescence quantum yields have been established in different solvents. Using UV-visible spectroscopy, dynamic light scattering, as well as transmission and confocal microscopy, aggregation of macrocycles was studied. It was evaluated that calixarene derivatives with alkyl substituents form spherical aggregates, while symmetrical tetrafluorescein-containing thiacalix[4]arene forms extended worm-like aggregates. The macrocycle containing tetradecyl fragments was found to be the most efficient in photoredox ipso-oxidation of phenylboronic acid. In addition, it was shown that in a number of different electron donors (NEt_3_, DABCO and iPr_2_EtN), the photoredox ipso-oxidation proceeds best with triethylamine. It has been shown that a low molecular weight surfactant Triton-X100 can also improve the photocatalytic abilities of an oligoethylene glycol fluorescein derivative, thus showing the importance of a combination of micellar and photoredox catalysis.

## 1. Introduction

Fluorescent molecules and materials have attracted the attention of researchers for a long time. Such interest is caused by the variety of applications of fluorescent molecules. The presence of a fluorescent unit in the molecule is a marker that allows anyone to track any changes that occur with the molecule itself. Due to the formation of exciplexes [1,2], photo-induced electron transfer [3,4] and other energy transfer processes [5,6], various fluorescent chemo/biosensors can be built. On the other hand, the presence of a fluorescent fragment makes it possible to use molecules for tracking in living systems, which is of great practical importance both in biology and medicine. Of particular interest are fluorescent derivatives of macrocycles, among which the leading place has been occupied by calixarenes–macrocycles with (almost) unlimited properties [7,8,9,10,11,12]. The introduction of fluorescent fragments into their composition makes it easy to visualize the processes of molecular recognition, due to which a number of extensive reviews are presented in the literature on fluorescent derivatives of calixarenes for chemo/biosensorics [13,14,15]. In the last decade, the revival of chemists’ interest in radical reactions initiated an avalanche of interest in another aspect of the use of fluorescent molecules—photoredox catalysis [16,17,18,19]. It is known that during catalytic transformations the macrocyclic cavity can act as a mimic of natural enzymes, concentrating the reagents or arranging them the right way with respect to each other [20]. Therefore, the combination of macrocycles with fluorescent molecules for photoredox catalysis looks very attractive [21,22,23]. Of particular interest are amphiphilic derivatives of fluorescent dyes. Giving amphiphilic properties to such systems makes it possible to obtain highly organized structures in aqueous media, which is convenient both from the point of view of chemosensors capable of operating in aqueous media and for photocatalysis of organic molecules in water, which can be solubilized by amphiphilic photoredox catalyst. We demonstrated that the most convenient way to synthesize amphiphilic derivatives of calix[4]arenes is the azide-alkyne cycloaddition (AAC) reaction of macrocyclic azides with functional polar alkynes [24,25,26]. In particular, using the click approach, our group recently obtained a fluorescein-calix[4]arene conjugate, which demonstrated excellent photocatalytic activity in photoredox oxidation of phenylboronic acid [27]. Continuing this research, herein we present the synthesis and properties of some new derivatives of classical calix[4]arene and thiacalix[4]arene containing polar oxyethyl fluorescein fragments.

## 2. Results and Discussion

### 2.1. Synthesis

**Scheme 1:**
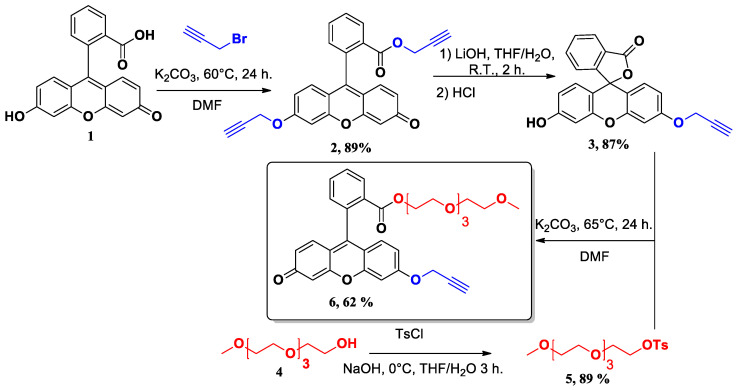
Synthetic pathway for fluorescein **6**.

To build target macrocyclic adducts with fluorescein fragments, the first stage of the work was the synthesis of a click chemistry precursor, a fluorescein derivative, which contains a terminal propargyl fragment and a tetraethylene glycol fragment to increase its water solubility. For this, o-propargylfluorescein **3**, obtained from fluorescein **1** by di-propargylation followed by ester hydrolysis with LiOH [28] was reacted with tosylate **5**, prepared from monomethyl ether of tetraethyleneglycol **4 [29]** to give fluoresceine derivative **6** with 62% yield (Scheme 1).

The structure of fluorescein **6** was well-proven using NMR ^1^H, ^13^C, IR and high-resolution ESI (HRESI) mass spectrometry (See Supplementary Materials Figure S1). On the ^1^H NMR spectrum, xanthene aromatic skeleton peaks are observed as a doublet of doublets at 8.28 ppm, 7.29 ppm, 6.79 ppm, 6.53 ppm; a doublet of triplets at 7.73 and 7.67 ppm; and as doublets at 7.06 ppm, 6.91 ppm and 6.86 ppm. The protons of the propargyl group appear as a doublet at 4.80 ppm and a triplet at 2.64 ppm. The ethylene protons of the methyltetraethylene glycol appear as a multiplet at 4.14 ppm, a singlet at 3.62 ppm and a set of triplets at 3.53, 3.45 and 3.40 ppm; methyl protons appear as a singlet at 3.36 ppm (See Supplementary Materials Figure S1a). According to the HRESI MS data, there is the presence of [M+H]+ ion (*m/z* calculated for [C_32_H_33_O_9_]^+^ 561.2119, found: 561.2112).

**Scheme 2:**
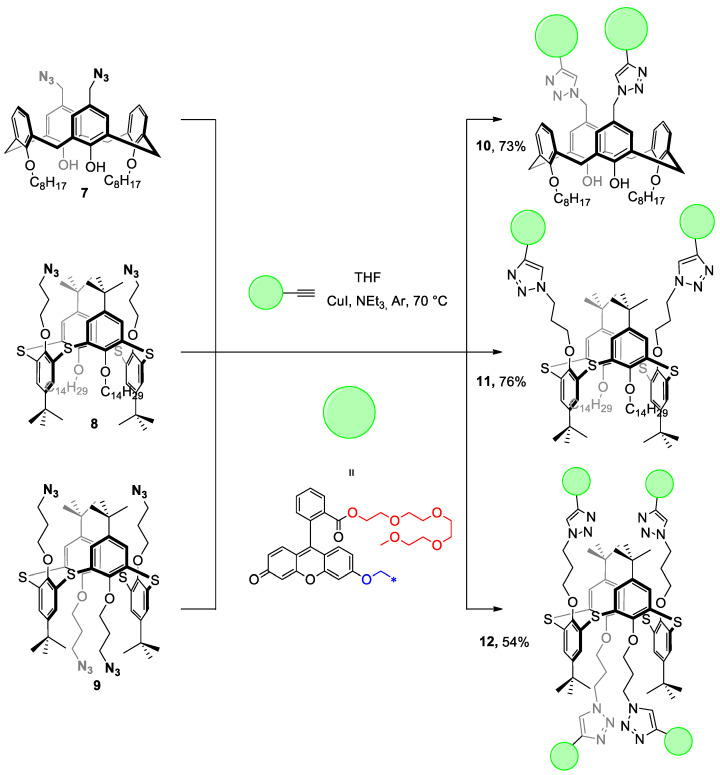
Synthesis of calix[4]arene–fluoresceine conjugates **10**–**12**.

The next step was the reaction of calix[4]arenes **7**–**9** containing different numbers of azide groups with the resulting fluorescein derivative **6** (Scheme 2). For the reaction, fluorescein **6** was used at the rate of 1 equivalent per 1 azide group of the macrocycle. Refluxing in THF in the presence of copper iodide turned out to be the most optimal for carrying out the CuAAC reaction. After 12–48 h compounds **10**–**12** were isolated as orange solids with yields from moderate to excellent. Compounds **11** and **12** were additionally purified using column chromatography.

The structure of all macrocycles was well proven using the same methods as for **6**. Thus, in the spectrum of classical calixarene **10** (See Supplementary Materials Figure S2a), besides the signals of the protons of fluoresceine fragments, new signals corresponding to two methylene fragments of the triazole ring appear at 5.38 and 5.24 ppm as well as a signal at 7.51 ppm, which corresponds to the aromatic proton of the triazole rings. The composition of the compound is precisely defined by HRESI MS (See Supplementary Materials Figure S2d): singly [M+H]^1+^ and doubly charged [M+2H]^2+^ cations were found with *m/z* 1880.8709 and 940.9387, respectively (theoretical values are 1880.8718 and 940.9395). Stereoisomeric configuration of macrocycle **10** was proved as a cone using a 2D NOESY experiment (See Supplementary Materials Figure S2e). The spectrum contains cross peaks between the protons of the phenol hydroxyl group with the protons of the methylene group of the octyloxy substituents on the lower rim (δ = 8.65 and 2.06 ppm, respectively), as well as cross peaks between the calixarene aromatic protons of adjacent benzene units (δ = 6.86 and 6.98 ppm, respectively). Other important cross peaks reflect the proximity of triazole protons with two methylene groups between the calixarene and fluorescein moieties (δ = 7.54 at 5.27 and 5.40 ppm). In the case of thiacalixarene **11**, the 1,3-alternate configuration was also confirmed using 2D NMR. The NOESY spectrum (See Supplementary Materials Figure S3e) shows cross peaks between the aromatic ring proton 25 of the calixarene platform and the corresponding tert-butyl fragment proton 26 (δ = 7.37 and 1.13 ppm, respectively), as well as cross peaks with methylene and methyl fragments of the tetradecyl substituent (δ = 7.37 and 1.30 ppm, respectively); the aromatic ring protons 27 have cross peaks with the methylene protons 22 of the propyl substituent (δ = 7.26 and 4.06 ppm, respectively), as well as with the protons 28 of the tert-butyl fragment (δ = 7.25 and 1.07 ppm, respectively); methylene protons 22 have cross peaks with protons of the tert-butyl fragment 28 (δ = 3.77 and 1.10 ppm, respectively), which unambiguously confirms the stereoisomeric form of macrocycle **11** as 1,3-alternate. Doubly charged cation [M+2H]^2+^ with *m/z* 1201.1024 (theoretical *m/z* = 1201.1028) was found in HRESI MS (See Supplementary Materials Figure S3d). The symmetrical structure of thiacalixarene **12** leads to a simplification of the NMR spectrum (See Supplementary Materials Figure S4a). Thus, in addition to the signals of the protons of the fluorescein fragments, the spectrum contains the only one signal of the aromatic protons of calixarene as a singlet at 7.26 ppm and an intense signal of tert-butyl protons as a singlet at 1.05. The triazole proton signal appears at 7.75 ppm. Triply charged cation [M+3H]^3+^ with *m/z* 1099.7649 (theoretical *m/z* = 1099.7654) was found in HRESI MS (See Supplementary Materials Figure S4d).

### 2.2. Photophysical Properties

The next step was the study of some photophysical properties of the synthesized macrocycles. The most important characteristic of fluorescent molecules is the fluorescent quantum yield, which is estimated as the ratio of emitted photons (through fluorescence) to the number of absorbed photons [30]. There are two options for estimating the quantum yield—the absolute and the relative method. The relative method is the simplest and requires only recording the absorption and emission spectra of unknown samples and a standard with a known quantum yield. Then a calibration graph of the integrated fluorescence intensity versus absorption is plotted, and the quantum yield is calculated by the gradient method [31] using Formula (1),

(1) $$Q_{s}=Q_{r}\left(\frac{m_{s}}{m_{r}}\right){\left(\frac{n_{s}}{n_{r}}\right)}^{2}$$

where $Q_{s}$ is the fluorescence quantum yield of unknown sample, $Q_{r}$ is the fluorescence quantum yield of known standard, $m_{s}$ and $m_{r}$ are the gradients of the plot of integrated fluorescence intensity against absorbance, $n_{s}$ and $n_{r}$ are the refractive indexes of the solvents used for unknown sample and standard, respectively. Taking into account that absolute fluorescein disodium salt quantum yield is 95% (0.1 M NaOH) [32,33], relative fluorescence quantum yields of staring alkyne **6** as well as compounds **10**–**12** were calculated from fluorescence and absorbance measurements (Table 1, See Supplementary Materials Figure S5).

**Figure 1:**
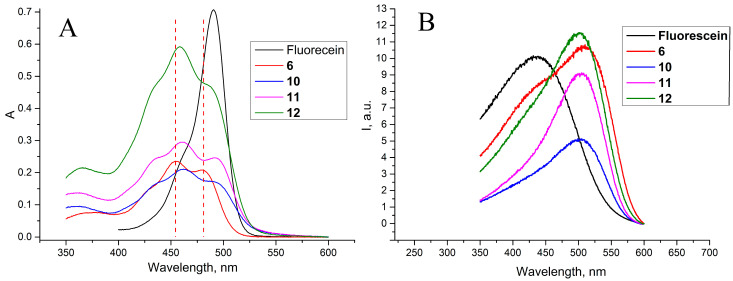
Absorption (**A**) and emission spectra of fluorescein (in 0.1 NaOH) and its derivatives **6**, **10** –**12** in water (1% DMF), C = 8.75 µM (for fluoresceine emission C = 2 µM).

According to the data obtained, the introduction of the alkynyl and ethoxyethyl fragment to compound **6** does not lead to a strong decrease in the quantum yield relative to unsubstituted fluorescein disodium salt in aqueous solution. The introduction of fluorescein fragments into the macrocyclic platform, on the contrary, leads to a noticeable decrease in the quantum yield. Considering that, like many other polyaromatic systems, fluorescein derivatives tend to form H or J-type aggregates [34,35,36], such a decrease in the quantum yield can be associated with the close location of two dye fragments on the same molecular platform. However, even this quantum yield (30%) is quite typical for ether-esters of fluorescein, which often have quantum yields around 30–40% in protic solvents [37,38]. It is known that the type of dye aggregates according to the Kascha theory [39] can be easily distinguished by the shift of the absorption maximum in the spectrum of the dimer with respect to the spectrum of the initial monomer—so in aggregates of the H-type (face-to-face type) a hypsochromic (blue) shift of the absorption maximum is observed, while formation of J-aggregates results in the bathochromic shift [40]. When analyzing the absorption spectra of the initial fluorescein **6**, characteristic absorption maxima are observed at 455 nm and 481 nm, as well as a shoulder at 429, typical for the open quinoid form of the dye [41,42] (Figure 1A).

**Figure 2:**
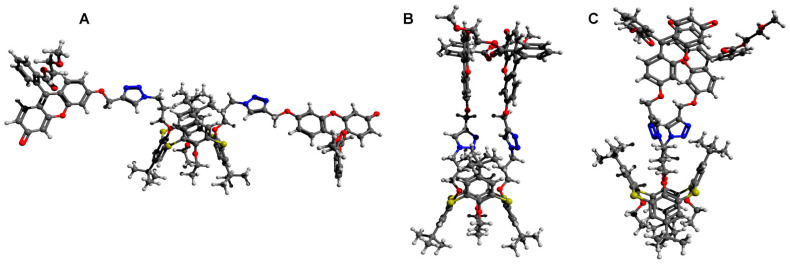
Structure of **11** conformer with spaced fluorescein fragments (**A**) and 11 conformer with stacked fluorescein fragments ((**B**,**C**), two-side view) according to the calculations (tetraethylene glycol and tetradecyl moieties are truncated for simplicity).

The introduction of florescein fragments into the macrocyclic platform leads to a bathochromic shift of bands at 455 and 480 nm, which is more pronounced in the case of di-substituted macrocycles **10** and **11** (∆λ is 9 nm) and less pronounced in the case of tetra-substituted macrocycle **12** (∆λ is 4 nm). An analysis of the fluorescence spectra (Figure 1B) in this case shows that the emission maximum at 514 nm upon the introduction of fluorescein fragments into the macrocycle, on the contrary, shifts hypochromically (∆λ is 9 nm). Correspondingly, the Stokes shift for calixarene derivatives becomes smaller, which is also characteristic of J-aggregates [43]. Taking into account that the observed shifts do not depend on the concentration, the formation of intramolecular J-aggregates is most probable. Quantum-chemical calculations were carried out to check the probability of formation of intra/intermolecular aggregates. Due to the size of the molecule, quantum-chemical calculations were done in the Priroda 19 package, which has one of the best scalabilities in time/system size. The difference in the Gibbs energy between the intramolecularly stacked (Figure 2B,C) and the open conformer form of calixarene (Figure 2A) is ~9 kcal/mol. Thus, the stacked form is the dominant conformer form in the gas phase and is highly probable in water.

It is noteworthy that on going to aprotic solvents, the quantum yield of the initial fluorescein decreases significantly (Table 1). It is known that hydrogen bonding in proton-donor solvents significantly affects the rate constant of internal conversion in fluorescein derivatives—in proton-donor solvents, the quantum yield is significantly higher than in aprotic ones [44]. At the same time, the quantum yield of both alkyne **6** itself and its macrocyclic derivatives in DMF lies in a close range. Upon transition to nonpolar toluene, the quantum yield of alkyne remains at the level of 23%, while the quantum yields of macrocycles decrease. This can be explained by the possible formation of reverse aggregates and the resulting concentration quenching.

### 2.3. Aggregation Abilities

Taking into account the amphiphilic nature of the synthesized macrocycles **10**–**12**, as well as the observed formation of presumably J-dimers, the aggregation of macrocycles in an aqueous solution containing 1% DMF was studied using UV-visible spectrometry and dynamic light scattering (DLS) method. To begin with, the absorption spectra of the obtained macrocycles were taken in a 1% DMF solution at different concentrations. It was found that when a certain concentration is reached, the ratio of the intensities of the absorption bands at 492 and 462 nm changes (See Supplementary Materials Figure S6), which is associated with a change in the environment of the molecules and indicate aggregation. For macrocycle **10**, the maximum change of absorption ratio was found at 1.9 µM, for macrocycle **11** at 1.4 µM and for tetra-fluorescein derivative **12** at 3 µM. A further increase in the concentration of the macrocycle up to a concentration of 50 µM did not change the absorption ratio. Obtained values lie within the typical values of the critical aggregation concentration for classical calix[4]arene [26] and thiacalix[4]arene [12] amphiphiles. Dynamic light scattering data for solutions containing 12.5 µM of studied calixarenes confirmed the formation of aggregates. Size measurement of the same systems at a concentration of 0.5 µM (below critical aggregation concentration) did not show the presence of stable particles in the solution. According to the data obtained (Table 2), indeed, all the studied systems form aggregates in aqueous solutions containing 1% DMF. By itself, DMF in an amount of 1% does not form any fixed aggregates. Macrocycle **10** containing octyl substituents forms submicron aggregates with an average hydrodynamic diameter of 260 nm and a rather low polydispersity index of 0.210. The more lipophilic tetradecyl-substituted calixarene **11** forms more compact aggregates with an average hydrodynamic diameter of 166 nm and the lowest polydispersity index in the series (0.144). Macrocycle **12**, having a symmetrical structure, also forms aggregates. However, in this case, a bimodal distribution of aggregates is observed—148 nm and 36 nm. Such a distribution may indicate the formation of extended aggregates of non-spherical morphology.

**Figure 3:**
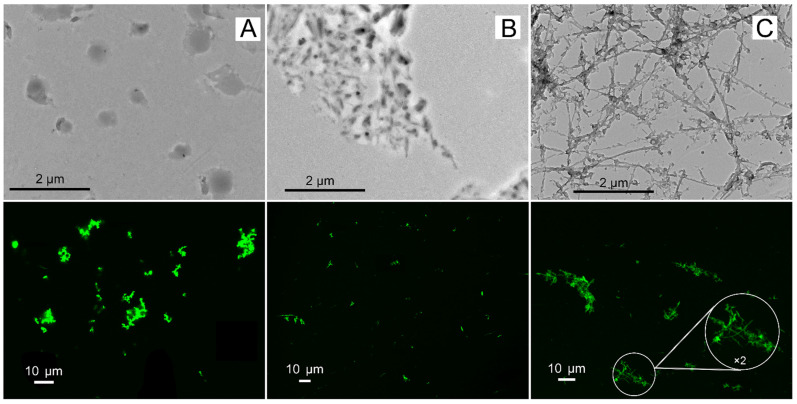
TEM and confocal microscopy images (λ_ex_ = 405 nm, 410–695 nm scan range) of aggregates formed by **10** (**A**), **11** (**B**) or **12** (**C**) in water (1% DMF), C (**10**–**12**) = 0.0125 mM.

The morphology of aggregates formed by macrocycles was also established using transmission electron microscopy (TEM) and confocal microscopy (Figure 3). According to the obtained microphotographs, macrocycle **10** forms large submicron aggregates up to 900 nm in size. These aggregates are clearly visible on a confocal microscope due to their intense fluorescence. Macrocycle **11**, on the contrary, forms nanoparticles with sizes of 100–150 nm. An interesting morphology was recorded in the case of compound **12**. The formation of worm/rod-like structures is observed, which, due to their emission, are also clearly visible on a confocal microscope.

The obtained picture correlates well with the DLS results of studying the sizes of aggregates in solution and confirms that the bimodal distribution of aggregates in the case of macrocycle **12** is a consequence of the formation of extended worm/rod-like aggregates. Additionally, the qualitative elemental composition of the aggregates formed by compound **11** was determined using energy-dispersive X-ray spectroscopy (EDX) (See Supplementary Materials Figure S7). Thus, according to the obtained data, the sample contains signals of carbon, hydrogen, nitrogen, oxygen and sulfur atoms as well as copper signals from copper-grid in full accordance with the composition of the macrocycle.

**Scheme 3:**
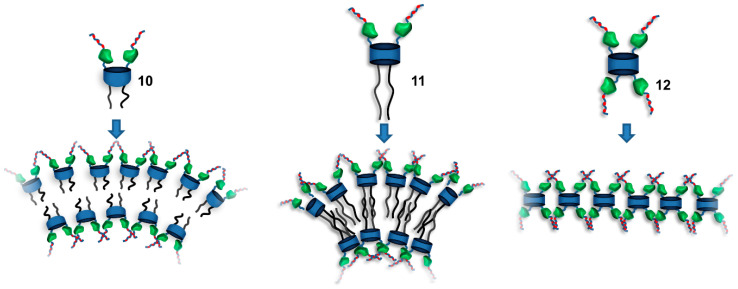
Synthesis of calix[4]arene–fluoresceine conjugates **10**–**12**.

Thus, the formation of aggregates can be schematically depicted as follows in Scheme 3. Compounds **10** and **11** form vesicle-like aggregates [45]. Compound **11** forms more compact aggregates, which is explained by more efficient hydrophobic interactions between long tetradecyl fragments. Compound **12** forms extended structures, the formation of which can involve both hydrophobic interactions of the calixarene backbone and π-π stacking interactions between electron-rich fluorescein residues.

### 2.4. Photocatalytic Activities

**Scheme 4:**
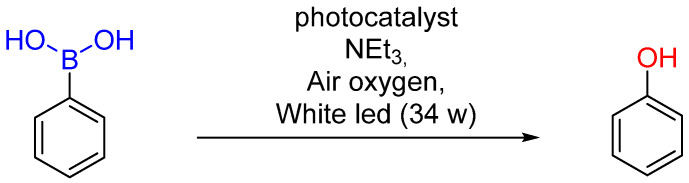
Model photoredox ipso oxidative hydroxylation of phenylboronic acid.

Photoredox ipso oxidative hydroxylation of phenylboronic acid is very convenient for testing the catalytic activity of photosensitizers in water due to the good solubility of both phenylboronic acids themselves and their oxidation products, phenols. This reaction is most often carried out using metal–containing photosensitizers such as [Ru(bpy)_3_]Cl_2_ [46] or Ir-based porous organic polymer [47], zinc phthalocyanines [48], copper-doped g-C_3_N_4_ [49], MOFs [50] and TiO_2_-based materials [51,52]. However, it was shown that organic photosensitizers, in particular those based on covalent organic frameworks (COFs) contining benzothiazole [53] or carbazole [54] moieties as well as dye-based photosensitizers as methylene blue [55], acridinium salts [56], acetophenone derivatives [57] or rose Bengal [58] are good metal-free alternative to act as single electron transfer (SET) agent in this reaction. As noted, our group recently demonstrated excellent photocatalytic activity in photo-redox oxidation of phenylboronic acid of fluorescein-calix[4]arene conjugate [27]. Thereby, compounds **10**–**12** as well as starting alkyne **6** were tested as photocatalysts in photoredox ipso oxidative hydroxylation of phenylboronic acid using air oxygen under white led light (Scheme 4). For this, a self-made photo irradiator was assembled based on an LED strip with a total power of 34 watts (See Supplementary Materials Figure S8). To remove heat excess, the photoreactor was cooled by water jacket; for air convection, a gap was specially left between the irradiator and the surface of the magnetic stirrer.

**Figure 4:**
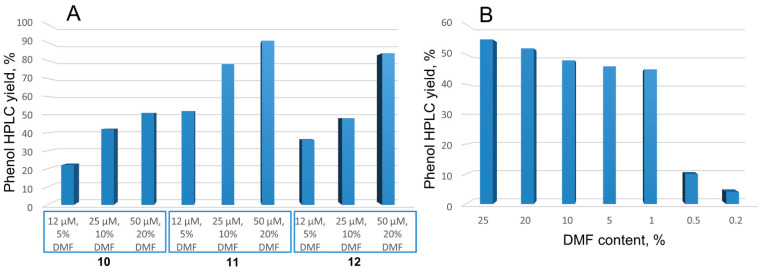
HPLC yield of phenol from photoredox ipso oxidative hydroxylation of phenylboronic acid using **10**–**12** after 10 h of irradiation (White LED, 34 W) at different loading and DMF content (**A**) and HPLC yield of phenol vs DMF content using **11**. C(PhB(OH_2_) = 10 mM, C (NEt_3_) = 20 mM, C (**10**–**12**) = 12–50 μM (**A**); C (**11**) = 12 μM.

HPLC-UV was used for quantitative control of the reaction using calibration curve constructed from known amounts of phenol. The reaction was carried out in a water–DMF mixture (total volume 0.5 mL) in the presence of various amounts of a catalyst. According to the data obtained (Figure 4A), with an increase in the amounts of both catalyst and DMF, the yield of phenol increases. The best results in all cases were demonstrated by macrocycle **11** with tetradecyl fragments. Thus, at a DMF content of 20% and a catalyst content of 50 µmol (0.5 mol % with respect to phenylboronic acid), it was possible to achieve 91% phenol content in the reaction mixture.

**Figure 5:**
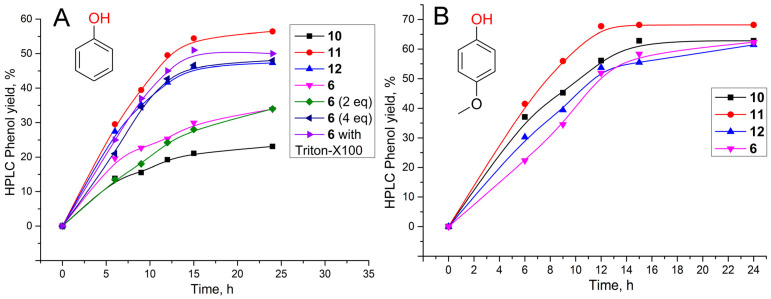
HPLC yield of phenol (**A**) or 4-methoxyphenol (**B**) from photoredox ipso oxidative hydroxylation of phenylboronic/4-methoxyphenyl boronic acid using **6** and **10**–**12** vs. time of irradiation (White LED, 34 W), 1% DMF content. C (PhB(OH_2_)) or (CH_3_C_6_H_4_B(OH_2_)) = 10 mM, C (NEt_3_) = 20 mM, C (**6**, **10**–**12**) = 12 μM, C (Triton X-100 = 0.3 mM).

The effect of the DMF content on the phenol yield was studied in more detail at a catalyst **11** concentration of 0.0125 mmol (which corresponds to 0.1 mol% of the catalyst) (Figure 4B). According to the data obtained, a decrease in the DMF content down to 1% does not lead to a significant decrease in the yield of phenol. A further decrease in the DMF content to 0.5% led to a decrease in the solubility of catalyst **11** and, as a consequence, a decrease in its activity. Therefore, it was decided to carry out further comparative studies with a catalyst content of 12 μM and a DMF content of 1%. According to the obtained data (Figure 5A), macrocycles **11** and **12** demonstrate the best results in the reaction with phenylboronic acid. Reactions performed without irradiation as well as in the presence of starting azides **8** and **9** without fluorescein fragments did not lead to products at all. In this case, the starting fluorescein **6** also exhibits moderate catalytic activity. It is worth returning to the quantum yield data. Despite the fact that fluorescein **6** has the highest quantum yield, it is inferior to systems based on calixarenes in photooxidation. Among calixarenes, the most hydrophobic macrocycle **11** is the best. Taking into account that **11** forms stable nanoaggregates with a low polydispersity index, in contrast to the less lipophilic macrocycle **10** and the symmetrical tetrafluorescein derivative **12**, most likely, it is the hydrophobic effect that plays the major role in increasing the catalytic activity. Just as in the case of classical micellar catalysis [59], most likely, phenylboric acid concentrates better in aggregates formed by the more lipophilic macrocycle **11**. A local increase in concentration, coupled with a close arrangement of electron transfer centers, leads to an increase of phenol HPLC yield.

It is noteworthy that when the more hydrophilic 4-methoxyphenol (ChemAxon calculated log P = 1.39 in contrast to phenylboronic acid’s log P = 1.64) is introduced into the reaction, the difference between the catalytic systems used becomes small (Figure 4B). This once again confirms that in the case of more hydrophobic phenol, the micellar catalytic effect takes place. It is noteworthy that two-fold increase in the content of the initial fluorescein **6** to 0.025 mM gives almost the same results as 0.0125 mM, while a fourfold increase to 0.05 mM allows one to obtain results similar to macrocycle **12** containing 4 fluorescein fragments. In order to prove the importance of the micellar effect on the increase in catalytic activity, it was decided to use the widely available non-ionic oxyethylated surfactant Triton-X100. When it was added to fluorescein **6** (0.3 mM Triton X-100 was used), the phenol yield increased significantly to the level of experiment with macrocycle **11**. Thus, the presence of a solubilizing surfactant is indeed capable of accelerating the photocatalytic reaction. The influence of different bases on the course of the reaction was studied (See Supplementary Materials Figure S9). It was found that the reaction proceeds most well with NEt_3_, while with DABCO and iPr_2_EtN the reaction proceeds noticeably worse. It is noteworthy that the data obtained in an analogous reaction in different publications vary greatly. Thus, in the article [49], in the same series of studied bases, triethylamine turned out to be better than diisopropylamine (29% and 13% of the product, respectively), but DABCO turned out to be generally ineffective. In the article [58], in a system using Rose bengal triethylamine and diisopropylethylamine were almost identical (95% and 92% of the product, respectively), and DABCO resulted in only 27% of the product. In contrast to these data, when using acetophenone derivative as a catalyst [57], triethylamine turned out to be worse than diisopropylethylamine (67 and 95% of the product, respectively). Taking into account one of the most convincing mechanisms of phenylboronic acid oxidation with the participation of ^18^O_2_, amine plays the role of a sacrificial electron donor, giving electrons to the photosensitizer with the following hydrogen atom transfer to give an iminium cation, which is then hydrolyzed to a secondary amine, which then again acts as a sacrificial electron donor [58,60]. It is noteworthy that when the nonlipophilic alkyne **6** is used in the reaction, the most effective base is iPr_2_EtN, while DABCO and NEt_3_ show the worst result (See Supplementary Materials Figure S8). Taking into account that in the series DABCO (log P = −0.13)–NEt_3_ (log P = 1.26)–iPr_2_EtN (log P = 2.09) the latter is the most hydrophobic, such a difference in the effect of iPr_2_EtN in reaction using lipophilic calixarene **11** can be explained by its location in the hydrophobic zone of the calixarene aggregates, which makes electron transfer to fluorescein residues less efficient. However, given the diverging literature data, the proposed assumption requires a more detailed study, including other amines and photoredox catalysts.

**Scheme 5:**
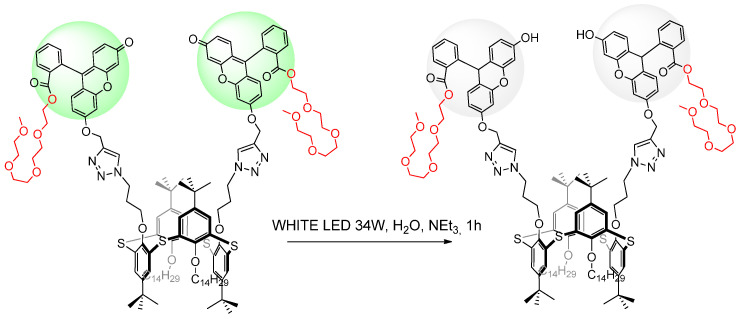
Photoredox reduction of **11** into leuco form.

Considering that compound **11** contains sulfur atoms, which can also undergo photooxidation to the corresponding sulfoxides and sulfones in the presence of electron-donating additives [61], an additional experiment on photoirradiation of compound **11** itself in the absence of a substrate (phenylboronic acid) to show the stability of the thiacalix[4]arene platform itself was carried out. For precise control, an HPLC-MS chromatogram of the **11** solution was taken before and after LED irradiation, which was carried out for 1 h (See Supplementary Materials Figure S10). According to the data obtained, we found HRESI MS signals corresponding to the addition of two hydrogen molecules to the macrocycle in the form of double charged cation [M+2H_2_+2H]^2+^ with *m/z* = 1203.1185 (calculated for C_138_H_184_N_6_O_22_S_4_^2+^ = 1203.1184) and two sets of signals related to double charged cations [M+2H_2_+Na+H]^2+^ with *m/z* = 1214.1090 (calculated for C_138_H_183_NaN_6_O_22_S_4_^2+^ = 1214.1094) and [M+2H_2_+2Na]^2+^ with *m/z* = 1225.0995 (calculated for C_138_H_184_N_6_O_22_S_4_^2+^ = 1225.1004) were detected in the high-resolution spectrum. Therefore, in the absence of a substrate, photocatalyst **11** underwent photoredox reduction of fluorescein fragments to a colorless leuco form (Scheme 5).

Photoreduction reaction of xanthene dyes in the presence of sacrificial amines has been known for quite a long time and proceeds through the formation of semiquinoid structures [62,63]. Amines, as well as in the process of photooxidation of phenylboronic acid, are oxidized during photoredox reduction of dye at the alpha carbon atom. Thus, the result obtained indicates the absence of photooxidation of the macrocyclic core itself. The observed transition of the dye incorporated into the macrocycle to the leuco form can be used in the future as a convenient synthetic approach to obtain macrocyclic fluorescent sensors capable of responding to oxidative stress due to the oxidation of leuco dye fragments with their subsequent emission [64].

## 3. Materials and Methods

### 3.1. Characterisation Methods

Chemicals were purchased from commercial suppliers and used as received. Solvents were purified according to standard procedures. Substance purity and the process of reaction were monitored by TLC on Merck UV 254 plates and visualized by exposure to UV with a VL-6.LC lamp (Vilber, Marne-la-Vallée, France).

^1^H and ^13^C NMR spectra as well as 2D ^1^H-^1^H NOESY were recorded on Bruker Avance 400 Nanobay (Bruker Corporation, Billerica, MA, USA) with signals from residual protons of DMSO-d_6_ or CDCl_3_ as internal standard.

The melting points were measured using the OIptimelt MPA100 melting point apparatus (Stanford Research Systems, Sunnyvale, CA, USA).

IR spectra in KBr pellets were recorded on a Bruker Vector-22 spectrometer (Bruker Corporation, Billerica, MA, USA). 

High-resolution mass spectra with electrospray ionization (HRESI MS) were obtained on an Agilent iFunnel 6550 Q-TOF LC/MS (Agilent Technologies, Santa Clara, CA, USA). Carrier gas: nitrogen, temperature 300 °C, carrier flow rate 12.l × min^−1^, nebulizer pressure 275 kPa, funnel voltage 3500 V, capillary voltage 500 V, total ion current recording mode, 100–3000 *m/z* mass range, scanning speed 7 spectra × s^−1^. HPLC-MS chromatograms were recorded using Agilent InfinityLab Poroshell 120 EC-C18 column (2.1 × 50 mm 1.9 Micron) (Agilent Technologies, Santa Clara, CA, USA) using 0.9 mL/min MeOH-H_2_O (90:20) eluent. 

HPLC–UV determination of phenol was performed on VWR Hitachi Chromaster HPLC system (Hitachi High-Tech Corporation, Tokyo, Japan), equipped with L-2130 pump, L-2400 UV detector and a 5310 column oven with Macherey-Nagel EC 250/4.6 NUCLEODUR C18 column (Macherey-Nagel GmbH, Duren, Germany) using 1.5 mL/min CH_3_CN-H_2_O (80:20) eluent. 

TEM was performed on Hitachi HT7700 ExaLens (Hitachi High-Tech Corporation, Tokyo, Japan) in Interdisciplinary Center for Analytical Microscopy of Kazan Federal University. The images were acquired at an accelerating voltage of 100 kV. Samples were ultrasonicated in water for 10 min, dispersed on 200 mesh copper grids with continuous formvar support films and then dried over 3 h. 

DLS and ELS experiments were carried out on Zetasizer Nano ZS instrument (Malvern Panalytical, Worcestershire, UK) with 4 mW 633 nm He–Ne laser light source and a light scattering angle of 173°. The data were treated with DTS software (Dispersion Technology Software 5.00). The solutions were filtered through 0.8 μM filter before the measurements to remove dust. The experiments were carried out in the disposable plastic cells DTS 0012 (size) or in the disposable folded capillary cells DTS 1070 (zeta potential) (Sigma–Aldrich, St. Louis, MO, USA) at 298K with at least three experiments for each system. 

Confocal laser microscopy images were obtained via CLMS on an inverted Carl Zeiss LSM 780 confocal laser-scanning microscope (Carl Zeiss, Jena, Germany). 

UV-visible spectra were recorded using a Shimadzu UV-2600 spectrophotometer equipped with a Shimadzu TCC-100 thermostat (Shimadzu Corporation, Kyoto, Japan).

Fluorescence spectra were performed in 10.0 mm quartz cuvettes and recorded on a Fluorolog FL-221 spectrofluorimeter (HORIBA Jobin Yvon, Kyoto, Japan) using excitation wavelength 430 nm with a 1 nm slit. All studies were conducted at 298 K.

### 3.2. Synthesis

o-Propargylfluorescein **3 [28]**, 2,5,8,11-tetraoxatridecan-13-ol tosylate **5 [29]**, 11,23-*Bis*(azidomethyl)-25,27-dihydroxy-26,28-dioctyloxycalix[4]arene **7** [27], 5,11,17,23-tetra-*tert*-butyl-25,27-ditetradecyloxy-26,28-di-3-azidopropyloxy-2,8,14,20-tetrathiacalix[4]arene **8 [65]**, and 5,11,17,23-tetra-*tert*-butyl-25,26,27,28-tetra*kis*(3- azidopropyloxy)-2,8,14,20-tetrathiacalix[4]arene **9** [66] were synthesized according to the literature procedures.

Synthesis of 2,5,8,11-tetraoxatridecan-13-yl 2-(3-oxo-6-(prop-2-yn-1-yloxy)-3H-xanthen-9-yl)benzoate (**6**). 

o-Propargylfluorescein (**3**) (0.98 g, 2.67 mmol), 2,5,8,11-tetraoxatridecan-13-ol tosylate (1.93 g, 5.34 mmol) and K_2_CO_3_ (0.89 g, 6.40 mmol) were dissolved in 10 mL of dry DMF and then stirred at 65 °C for 24 h. The completion of the reaction was determined via TLC (EtOAc with 1% MeOH). Afterward, the reaction mixture was diluted with 5 mL H_2_O and extracted by ethyl acetate (3 × 15 mL). Then organic layer was washed with brine (5 × 15 mL) and dried over anhydrous MgSO_4_. Solvent was evaporated to give **6** as a red oil, which was purified via SiO_2_ column chromatography (EtOAc) to afford compound (**2**) as an orange solid. Yield 0.72 g (62%). TLC R_f_ = 0.21 (EtOAc with 1% MeOH); mp 95 ℃.

^1^H NMR (400 MHz, CDCl_3_,*,* 25 °C) δ 8.28 (dd, *J* = 7.8, 1.4 Hz, 1H, ArH), 7.73 (td, *J* = 7.5, 1.5 Hz, 1H, ArH), 7.67 (td, *J* = 7.7, 1.4 Hz, 1H, ArH), 7.29 (dd, *J* = 7.5, 1.4 Hz, 1H, ArH), 7.06 (d, *J* = 2.4 Hz, 1H, ArH), 6.91 (d, *J* = 8.9 Hz, 1H, ArH), 6.86 (d, *J* = 9.7 Hz, 1H, ArH), 6.79 (dd, *J* = 8.9, 2.5 Hz, 1H, ArH), 6.53 (dd, *J* = 9.8, 1.9 Hz, 1H, ArH), 6.44 (d, *J* = 1.9 Hz, 1H, ArH), 4.80 (d, *J* = 2.3 Hz, 2H, -CH_2_-C≡CH), 4.17–4.11 (m, 2H, -C(O)O-CH_2_-), 3.62 (s, 6H, -(CH_2_)_3_-), 3.53 (t, *J* = 5.5 Hz, 4H, -(CH_2_)_2_-), 3.45 (t, *J* = 5.2 Hz, 2H, -CH_2_-OCH_3_), 3.40 (t, *J* = 4.9 Hz, 2H, -CH_2_-), 3.36 (s, 3H, -CH_2_O-CH_3_), 2.64 (t, *J* = 2.3 Hz, 1H, -C≡CH). 

^13^C NMR (101 MHz, CDCl_3_, 25 °C) δ 185.79, 165.36, 161.76, 158.99, 156.12, 154.04, 150.01, 134.40, 132.87, 131.57, 130.58, 130.45, 130.39, 130.15, 129.81, 129.15, 118.10, 115.60, 113.75, 105.90, 101.60, 77.29, 77.09, 71.99, 70.64, 70.58, 70.55, 70.48, 68.72, 64.68, 59.14, 56.44.

IR (KBr) ν_max_ cm^−1^: 3232 (≡C–H), 2874 (C-H_Ar_), 2127 (C≡C), 1724 (C=O), 1601 (C_Ar_=C_Ar_), 1516 (C_Ar_=C_Ar_), 1278 (C-O), 1256 (C-O), 1106 (C-O).

HRESI MS (*m/z*) [M+H]^+^: calculated for [C_32_H_33_O_9_]^+^: 561.2119, found: 561.2112.

General procedure for the synthesis of compounds (**10–12**).

2,5,8,11-tetraoxatridecan-13-yl 2-(3-oxo-6-(prop-2-yn-1-yloxy)-*3H*-xanthen-9-yl)benzoate (**6**) was dissolved in 10 mL of dry THF, then NEt_3_ and CuI were added under an inert atmosphere. The resulting mixture was stirred for 5 min. After azides (**7**–**10**) were added. The reaction mixture was stirred at 70 °C for 12–48 h. The completion of the reaction was determined via TLC. Solvent was evaporated, and the residue was dissolved in chloroform and washed with sat. ammonia solution (3 × 20 mL), 1M HCl (3 × 15 mL) and H_2_O (1 × 15 mL), dried over anhydrous MgSO_4_, filtered off and concentrated under reduced pressure. 

Compound (**10**). Following the general procedure and using 2,5,8,11-tetraoxatridecan-13-yl-2-(3-oxo-6-(prop-2-yn-1-yloxy)-*3H*-xanthen-9-yl)benzoate (**6**) (0.17 g, 0.3 mmol), azide (**7**) (0.11 g, 0.15 mmol), CuI (0.003 g, 0.015 mmol) and NEt_3_ (1 mL), the substance was obtained as an orange solid. Yield 0.20 g (73%). TLC R_f_ = 0.19 (EtOAc with 20% MeOH); mp 83 ℃.

^1^H NMR (400 MHz, CDCl_3_,*,* 25 °C) δ 8.63 (s, 2H, OH_cal_), 8.27 (d, *J* = 7.8 Hz, 2H, ArH_Flu_), 7.72 (t, *J* = 7.3 Hz, 2H, ArH_Flu_), 7.66 (t, *J* = 7.6 Hz, 2H, ArH_Flu_), 7.51 (s, 2H, Trz), 7.27 (d, *J* = 6.4 Hz, 2H, ArH_Flu_), 7.07 (d, *J* = 2.5 Hz, 2H, ArH_Flu_), 7.01 (s, 4H, ArH_cal_), 6.91–6.83 (m, 8H, ArH_cal_ + ArH_Flu_), 6.80 (dd, *J* = 9.1, 2.3 Hz, 2H, ArH_Flu_), 6.75 (t, *J* = 7.5 Hz, 2H, ArH_cal_), 6.52 (dd, *J* = 9.8, 1.9 Hz, 2H, ArH_Flu_), 6.43 (s, 2H, ArH_Flu_), 5.38 (s, 4H, Flu-O-CH_2_-), 5.24 (s, 4H, -O-CH_2_-Trz), 4.27 (d, *J* = 12.8 Hz, 4H, Ar-CH_2_-Ar), 4.20–4.06 (m, 4H, -O-CH_2_-), 3.97 (t, *J* = 6.6 Hz, 4H, Ar-OCH_2_-), 3.65–3.59 (m, 12H, -CH_2_-), 3.53 (t, *J* = 4.7 Hz, 8H, -CH_2_-), 3.46 (t, *J* = 5.9, 3.7 Hz, 4H, -CH_2_-), 3.41 (t, *J* = 6.1, 3.0 Hz, 4H, -CH_2_-), 3.35–3.34 (m, 10H, -CH_2_O-CH_3_ + Ar-CH_2_-Ar), 2.04 (p, *J* = 6.9 Hz, 4H, -CH_2_-), 1.67 (p, *J* = 7.5 Hz, 4H, -CH_2_-), 1.47–1.28 (m, 16H, -CH_2_-), 0.88 (t, *J* = 6.5 Hz, 6H, -CH_2_-CH_3_).

^13^C NMR (101 MHz, CDCl_3_, 25 °C) δ 185.75, 165.33, 162.62, 159.01, 154.09, 152.05, 150.11, 134.45, 133.15, 132.84, 131.52, 130.59, 130.45, 130.37, 129.79, 129.17, 129.10, 128.95, 128.74, 125.55, 124.47, 123.05, 117.93, 115.36, 113.64, 105.88, 101.51, 77.14, 71.99, 70.63, 70.56, 70.50, 68.74, 64.66, 62.51, 59.11, 54.18, 36.57, 32.04, 31.47, 30.09, 29.80, 29.58, 29.43, 26.07, 22.79, 14.24.

IR (KBr) ν_max_ cm^−1^: 3295 (br, OH), 2924 (CH_3_), 2866 (CH_2_), 1720 (C=O_Flu_), 1644 (C-O_Flu_), 1520 (C_Ar_=C_Ar_), 1457 (C_Ar_=C_Ar_), 1284 (C-O), 1253(C-O), 1207, 1107 (C-O_TEG_).

HRESI MS (*m/z*) [M+H]^+^: calculated for [C_110_H_123_N_6_O_22_]^+^: 1880.8718, found: 1880.8709; [M+2H]^2+^: calcd. for [C_110_H_124_N_6_O_22_]^2+^: 940.9395, found: 940.9387.

Compound (**11**): Following the general procedure and using 2,5,8,11-tetraoxatridecan-13-yl-2-(3-oxo-6-(prop-2-yn-1-yloxy)-*3H*-xanthen-9-yl)benzoate (**6**) (0.11 g, 0.2 mmol), azide (**8**) (0.13 g, 0.1 mmol), CuI (0.002 g, 0.01 mmol) and NEt_3_ (1 mL), the crude product was purified via column chromatography (SiO_2_, EtOAc with 5% MeOH) to afford compound (**11**) as an orange solid. Yield 0.18 g (76%). TLC R_f_ = 0.39 (EtOAc with 20% MeOH). mp 113 °C (decomp.).

^1^H NMR (400 MHz, CDCl_3_*,* 25 °C) δ 8.27 (d, *J* = 7.8 Hz, 2H, ArH_Flu_), 7.78–7.69 (m, 4H, ArH_Flu_ + Trz), 7.66 (t, *J* = 7.6 Hz, 2H, ArH_Flu_), 7.34 (s, 4H, ArH_cal_), 7.29 (d, *J* = 4.3 Hz, 2H, ArH_Flu_), 7.26 (s, 4H, ArH_cal_), 7.21 (d, *J* = 2.2 Hz, 2H, ArH_Flu_), 6.89–6.80 (m, 6H, ArH_Flu_), 6.51 (d, *J* = 9.7 Hz, 2H, ArH_Flu_), 6.38 (dd, *J* = 9.0, 2.3 Hz, 2H, ArH_Flu_), 5.28 (s, 4H, Flu-O-CH_2_-), 4.22–4.06 (m, 4H, -CH_2_-), 4.02 (t, *J* = 6.8 Hz, 4H, Trz-CH_2_-CH_2_-), 3.75 (t, *J* = 6.7 Hz, 4H, -CH_2_-CH_2_-OCal), 3.62 (s, 12H, -CH_2_-), 3.55–3.52 (m, 8H, -CH_2_-), 3.51–3.40 (m, 8H, -CH_2_-), 3.35 (s, 6H, -CH_2_O-CH_3_), 1.76 (t, *J* = 7.0 Hz, 4H, Trz-CH_2_-CH_2_-), 1.27 (s, 70H, *t*-Bu + -(CH_2_)_13_-), 1.05 (s, 18H, *t*-Bu), 0.88 (t, *J* = 6.6 Hz, 6H, -(CH_2_)_13_-CH_3_).

^13^C NMR (101 MHz, CDCl_3_, 25 °C) δ 185.69, 165.23, 162.60, 159.03, 156.99, 154.15, 150.49, 145.86, 145.43, 134.36, 132.84, 131.47, 130.52, 130.24, 129.77, 129.20, 128.63, 127.82, 127.23, 123.27, 117.82, 115.34, 113.67, 105.74, 101.41, 72.67, 71.98, 70.54, 70.47, 70.44, 70.22, 68.98, 68.69, 68.10, 64.59, 62.50, 61.72, 59.04, 50.26, 34.26, 31.97, 31.48, 31.33, 30.01, 29.73, 29.42, 28.82, 26.82, 26.22, 25.80, 22.73, 14.17. 

IR (KBr) ν_max_ cm^−1^: 2920 (CH_3_), 2853 (CH_2_), 1721 (C = O_Flu_), 1641(C-O_Flu_), 1600 (C_ArH_ = C_ArH_), 1596 (C_ArH_ = C_ArH_), 1270 (C-O_ester_), 1257 (C-O _ester_), 1112 (C-O_TEG_).

HRESI MS (*m/z*) [M+2H]^2+^: calculatedfor [C_138_H_180_N_6_O_22_S_4_]^2+^: 1201.1028, found: 1201.1024.

Compound (**12**): Following the general procedure and using 2,5,8,11-tetraoxatridecan-13-yl-2-(3-oxo-6-(prop-2-yn-1-yloxy)-*3H*-xanthen-9-yl)benzoate (**6**) (0.15 g, 0.3 mmol), azide (**9**) (0.07 g, 0.07 mmol), CuI (0.001 g, 0.007 mmol) and NEt_3_ (1 mL), the crude was purified by column chromatography (SiO_2_, EtOAc with 5% MeOH) to afford compound (**12**) as an orange solid. Yield 0.12 g (54%). TLC R_f_ = 0.37 (EtOAc with 20% MeOH). mp 104 °C (decomp.).

^1^H NMR (400 MHz, CDCl_3_*,* 25 °C) δ 8.27 (d, *J* = 7.4 Hz, 4H, ArH_Flu_), 7.75 (s, 4H, Trz), 7.71 (t, *J* = 8.0 Hz, 4H, ArH_Flu_), 7.65 (t, *J* = 7.4 Hz, 4H, ArH_Flu_), 7.26 (s, 8H, ArH_cal_), 7.22 (d, *J* = 3.8 Hz, 4H, ArH_Flu_), 7.08–7.05 (m, 4H, ArH_Flu_), 6.91–6.81 (m, 6H, ArH_Flu_), 6.51 (d, *J* = 10.1 Hz, 4H, ArH_Flu_), 6.38 (dd, *J* = 6.7, 2.0 Hz, 4H, ArH_Flu_), 5.28 (s, 8H, Flu-O-CH_2_-), 4.20–4.05 (m, 16H, Trz-CH_2_-CH_2_- + -CH_2_-O-), 4.00–3.91 (m, 8H, -CH_2_-O-Cal), 3.61 (s, 24H, -CH_2_-), 3.57–3.50 (m, 16H, -CH_2_-), 3.50–3.42 (m, 16H, -CH_2_-), 3.35 (s, 12H, -CH_2_-O-CH_3_), 1.74–1.62 (m, 8H, Trz-CH_2_-CH_2_-), 1.05 (s, 36H, *t*-Bu).

^13^C NMR (101 MHz, CDCl_3_, 25 °C) 185.70, 165.23, 162.65, 161.82, 159.05, 156.36, 154.19, 150.43, 146.62, 142.59, 134.39, 132.88, 131.50, 130.54, 130.30, 129.82, 129.25, 129.17, 128.28, 127.46, 123.58, 117.86, 115.38, 113.78, 105.73, 101.61, 101.51, 77.16, 71.95, 70.59, 70.51, 68.74, 64.62, 62.45, 59.07, 56.43, 47.91, 34.28, 31.41, 31.14, 29.95.

IR (KBr) ν_max_ cm^−1^: 2959 (CH_3_), 2869 (CH_2_), 1722 (C=O_Flu_), 1643 (C-O_Flu_), 1598 (C_ArH_=C_ArH_), 1520 (C_ArH_=C_ArH_), 1266 (C-O_ester_), 1255 (C-O_ester_), 1108 (C-O_TEG_).

HRESI MS (*m/z*) [M+3H]^3+^: calculated for [C_180_H_201_N_12_O_40_S_4_]^3+^: 1099.7654, found: 1099.7649.

### 3.3. Photocatalytic Oxidation of Phenylboronic Acid

In a glass vial 10 mM of phenylboronic acid, 20 mM of triethylamine (or other used bases) and 1 mL of 1% DMF-water solution containing 12 μM photocatalyst were mixed together. The mixture was kept under white LED irradiation (34 W) bubbling with air for 10–24 h.

### 3.4. Quantum-Chemical Calculations

DFT calculations of calixarene **11** conformers were performed with Priroda 19 [67]. PBE level of theory with L2 basis set [68] (similar to cc-pVTZ [69]) in the gas phase was used. Conformer searching was carried out from multiple different initial geometries obtained from MMFF94 [70] optimized in Avogadro [71] structures.

## 4. Conclusions

A fluorescein click precursor containing a polar oligoethylene glycol fragment and a propargyl group was obtained for the first time. The resulting fluorescein was introduced into three different azide derivatives of calixarene: bis-azide based on classical calix[4]arene in the cone configuration and bis- and tetra-azides based on thiacalix[4]arene in the 1,3-alternate configuration. For the resulting fluorescein and its macrocyclic derivatives, the quantum yields of luminescence in different solvents were established. It was found that for the initial fluorescein, the maximum quantum yield (80%) is achieved in water, while in the case of macrocycles in water (1% DMF) and pure DMF systems, the quantum yield is about 30%. In toluene it drops to 15%. Using dynamic light scattering as well as transmission and confocal microscopy, it was shown that calixarene derivatives form aggregates of various morphologies in water (1% DMF). Thus, derivatives with alkyl substituents form spherical aggregates, while symmetrical tetrafluorescein-containing thiacalixarene forms extended worm-like aggregates. All the obtained systems were studied in the catalysis of photoredox ipso-oxidation of phenylboronic acid. The macrocycle containing tetradecyl fragments was found to be the most efficient in catalysis using triethylamine as a base. Most likely, the increased activity of the macrocycle is associated with its greater hydrophobicity, whereby it is able to act as an effective micellar catalyst, concentrating the substrate in the hydrophobic zone of the aggregates. Indeed, addition of widely used surfactant (Triton–X 100) to fluorescein click precursor led to an increase in conversion, proving thus the importance of micellar catalysis in accelerating the photooxidative ipso-oxidation of phenylboronic acid. The photostability of the thiacalix[4]arene-fluoresceine conjugate was evaluated in the absence of a substrate. Photoredox reduction of fluorescein fragments to a colorless leuco form was found, which is very promising for the synthesis of sensors capable of oxidative stress probing. 

## Figures and Tables

**Table 1:** Relative fluorescence quantum yield for different fluorescein derivatives against fluorescein disodium salt ^1^.

System	$Q_{s}$, % (Water with 1% Dimethylformamide (DMF))	$Q_{s}$, % (DMF)	$Q_{s}$, % (Toluene)
**6**	80.3	24.0	23.2
**1** **0**	30.2	31.1	7.8
**1** **1**	27.9	34.3	15.8
**1** **2**	28.8	29.5	14.9

^1^ Fluorescence and absorbance spectra were recorded in 10 mm quartz cell in the range of 2.5–20 µM.

**Table 2:** **^1^** DLS data for **10**–**12** aqueous solutions (containing 1% DMF).

System	D Average, nm	d1, nm	d2, nm	PDI
**10**	260 ± 22	258 ± 56 (100%)		0.210 ± 0.091
**11**	166 ± 1	177 ± 8 (100%)		0.144 ± 0.021
**12**	89 ± 2	148 ± 10 (75 ± 4%)	36 ± 2 (25 ± 4%)	0.390 ± 0.006

^1^ C (**10–12**) = 0.0125 mM.

## Data Availability

Not applicable.

## Supplementary Materials

The following supporting information can be downloaded at: https://www.mdpi.com/article/10.3390/molecules28010261/s1, Figure S1: NMR ^1^H (a), ^13^C (b), FT IR (c) and HRESI MS (d) spectra of 2,5,8,11-tetraoxatridecan-13-yl 2-(3-oxo-6-(prop-2-yn-1-yloxy)-3H-xanthen-9-yl)benzoate (**6**); Figure S2: NMR ^1^H (a), ^13^C (b), FT IR (c), HRESI MS (d) and 2D NOESY NMR ^1^H-^1^H (CDCl_3_) (e) spectra of compound (**10**). Figure S3. NMR ^1^H (a), ^13^C (b), FT IR (c), HRESI MS (d) and 2D NOESY NMR ^1^H-^1^H (CDCl_3_) (e) spectra of compound (**11**); Figure S4: NMR ^1^H (a), ^13^C (b), FT IR (c) and HRESI MS (d) spectra of compound (**12**); Figure S5: Integrated fluorescence intensity vs. max. absorbance at different concentrations for fluorescein (0.1M NaOH) and compounds **6**, **10**, **11**, **12** (water with 1% DMF, pure DMF or toluene); C = 2.5–20 µM; Figure S6: Ratio of absorption maxima at 492 and 462 nm vs. calixarene 10, 11, 12 concentration (water with 1% DMF); C = 0.0005–0.015 mM; Figure S7: EDX spectrum of aggregates formed by **11**; Figure S8: Photoreactor (white LED, 34 W); Figure S9: HPLC yield of phenol from photoredox ipso oxidative hydroxylation of phenylboronic acid using **6** and **11** vs. time of irradiation (White LED, 34 W), 1% DMF content with different bases C(PhB(OH_2_) = 10 mM, C (base) = 20 mM, C (**6**, **11**) = 12 μM (A); Figure S10: HPLC chromatogram and corresponding HRESI mass-spectra of **11** (0.0125 mM aqueous solution with 1% DMF) before (green) and after (blue) irradiation for 1 h in the presence of NEt_3_ (20 mM).

## Sample Availability

Samples of the compounds **6**, **10**–**12** are available from the authors.
