# Supplementary material for: Oxyethylated Fluoresceine—(thia)calix[4]arene Conjugates: Synthesis and Visible-Light Photoredox Catalysis in Water–Organic Media
Source: Molecules. 2022 Dec 28;28(1):261. doi: 10.3390/molecules28010261 (PMC9821991; doi:10.3390/molecules28010261)
Supplement: Supplementary file 1 [file molecules-28-00261-s001.zip › molecules-2098071-supplementary.pdf]

Oxyethylated fluoresceine — (thia)calix[4]arene conjugates: synthesis and visible-light photoredox catalysis in water-organic media.

Vladimir Burilov[1*], Aigul Fatykhova[1], Diana Mironova[1], Elsa Sultanova[1], Ramil Nugmanov[1], Alina Artemenko[1], Anastasia Volodina[1], Amina Daminova[2], Vladimir Evtugyn[2], Svetlana Solovieva[3] and Igor Antipin[1]

1. Alexander Butlerov Institute of Chemistry, Kazan Federal University, 18 Kremlevskaya st., 420008 Kazan, Russia.
2. Interdisciplinary Center for Analytical Microscopy, Kazan Federal University, 18 Kremlevskaya st., 420008 Kazan, Russia.
3. Arbuzov Institute of Organic and Physical Chemistry, FRC Kazan Scientific Center of RAS, 8 Arbuzov str., 420008 Kazan, Russia.

* Correspondence: ultrav@bk.ru, Tel.: (+7-843-2337344)

(a)

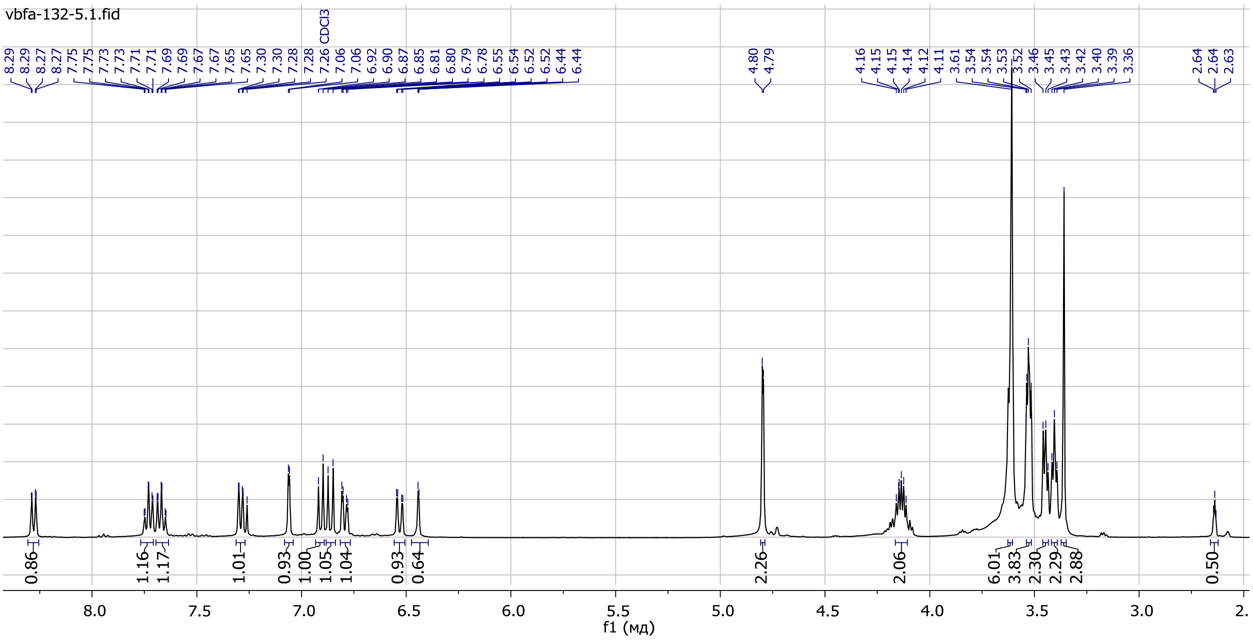
vbfa-132-5.1.fid
CDCl3
f1 (мд)

(b)

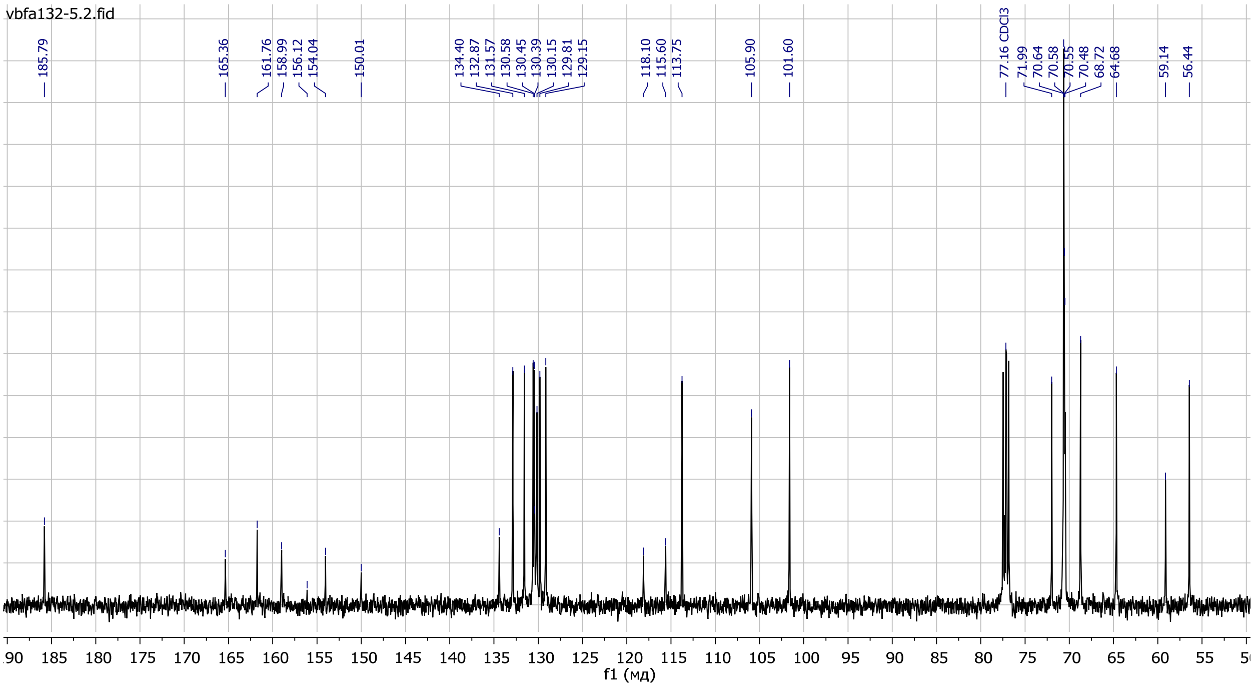
vbfa132-5.2.fid
185.79
165.36
161.76
158.99
156.12
154.04
150.01
134.40
132.87
131.57
130.58
130.45
130.39
130.15
129.81
129.15
118.10
115.60
113.75
105.90
101.60
77.16 CDCl3
71.99
70.64
70.58
70.55
70.48
68.72
64.68
59.14
56.44
f1 (мд)

(c)

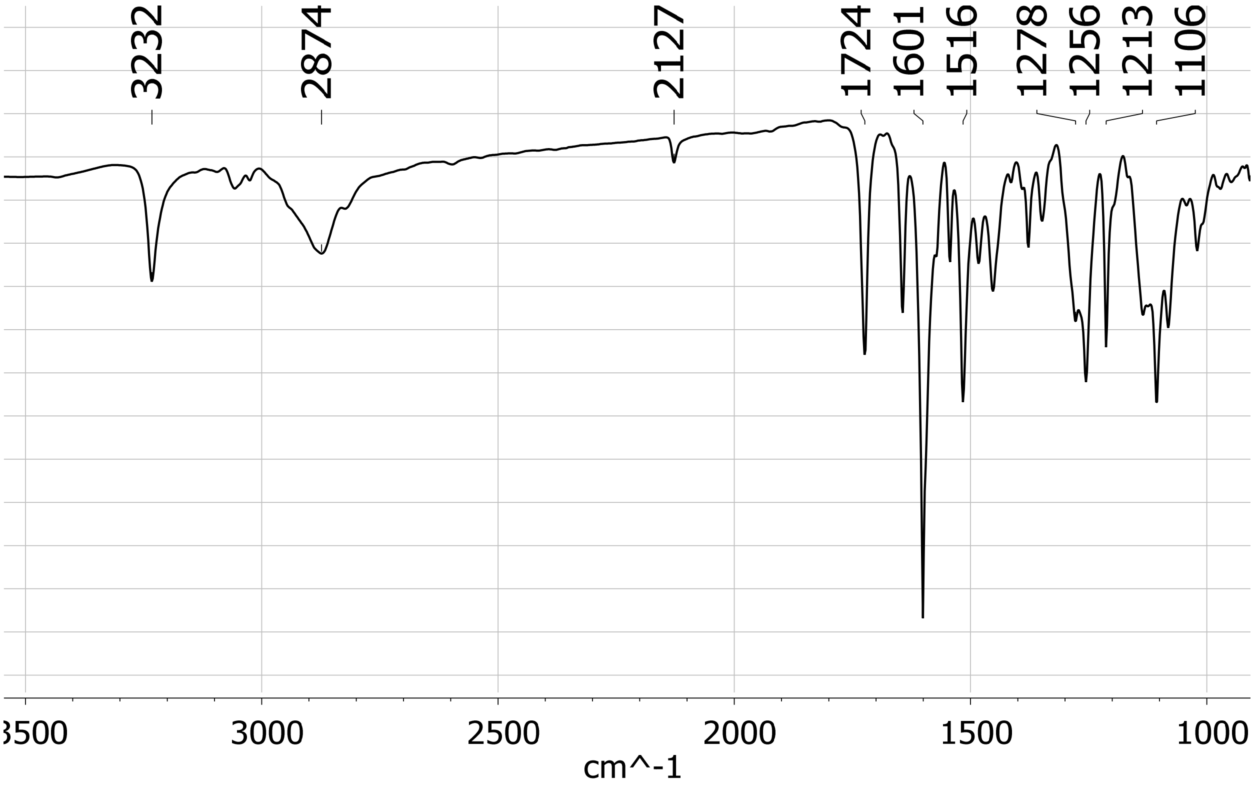

(d)

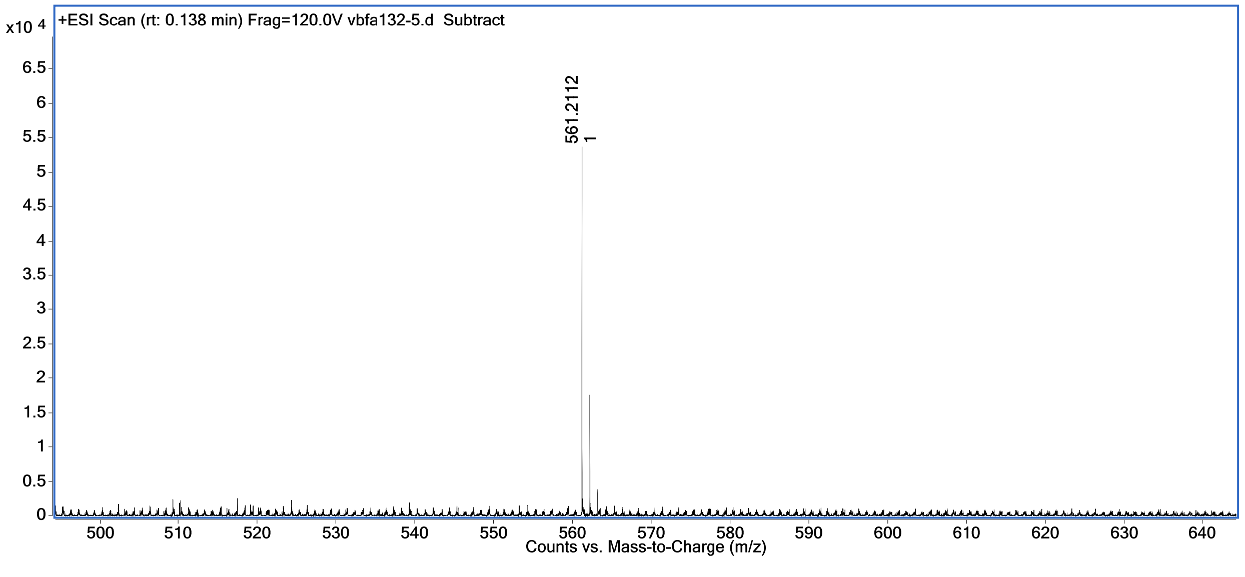

Figure S1. NMR ^{1}H (a), ^{13}C (b), FT IR (c) and HRESI MS (d) spectra of *2,5,8,11-tetraoxatridecan-13-yl 2-(3-oxo-6-(prop-2-yn-1-yloxy)-3H-xanthen-9-yl)benzoate* ***(6)***.

(a)

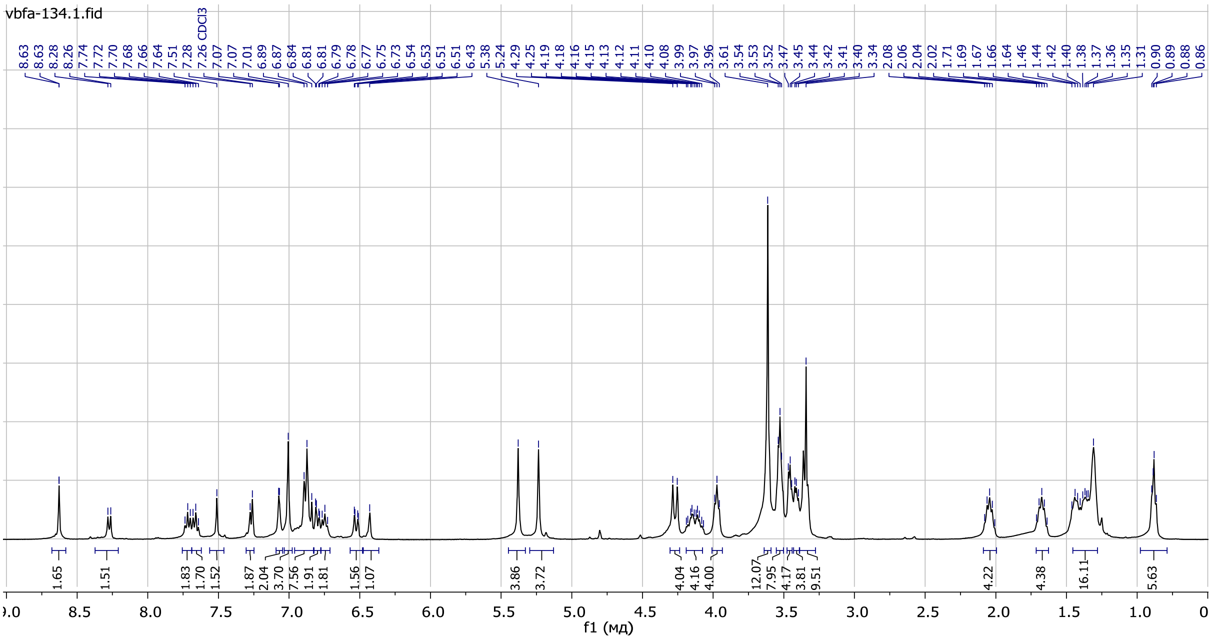

(b)

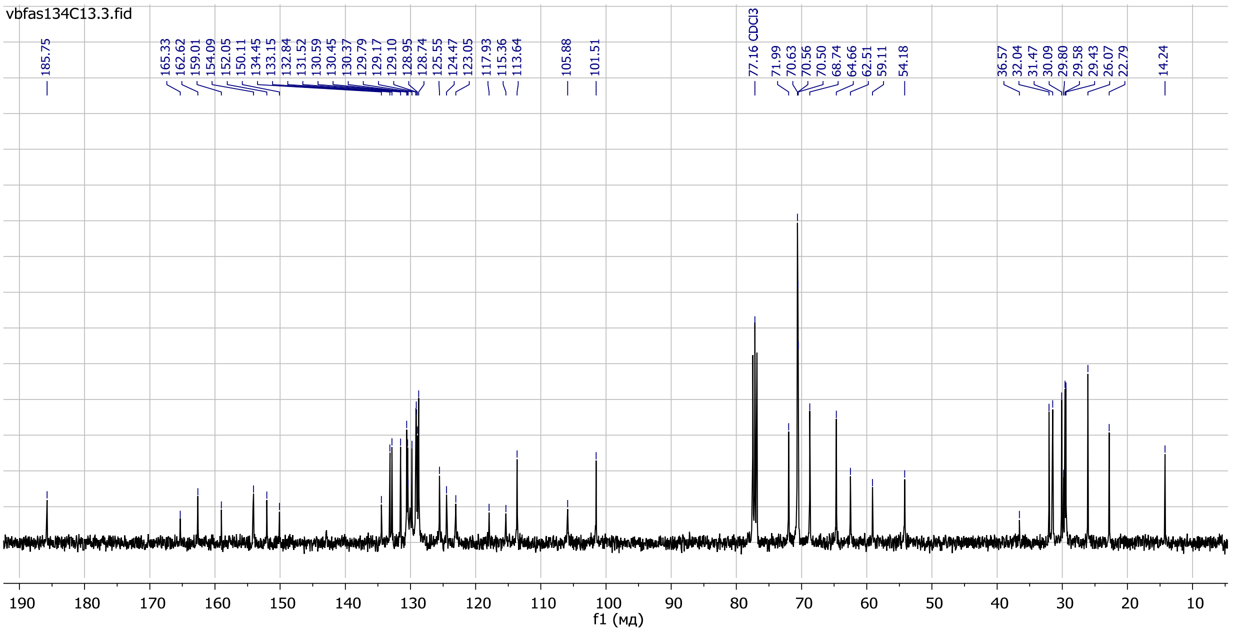

(c)

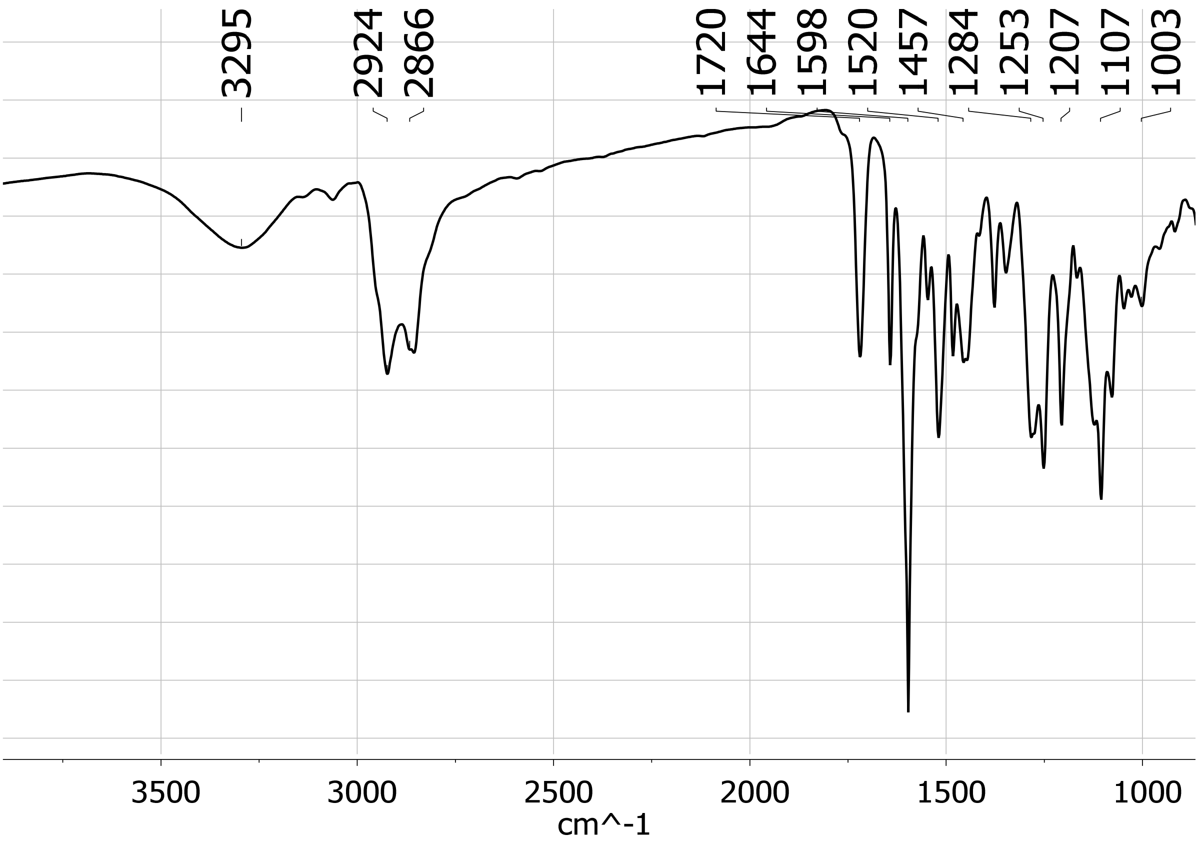
3295
2924
2866
1720
1644
1598
1520
1457
1284
1253
1207
1107
1003
3500
3000
2500
2000
1500
1000
cm^-1

(d)

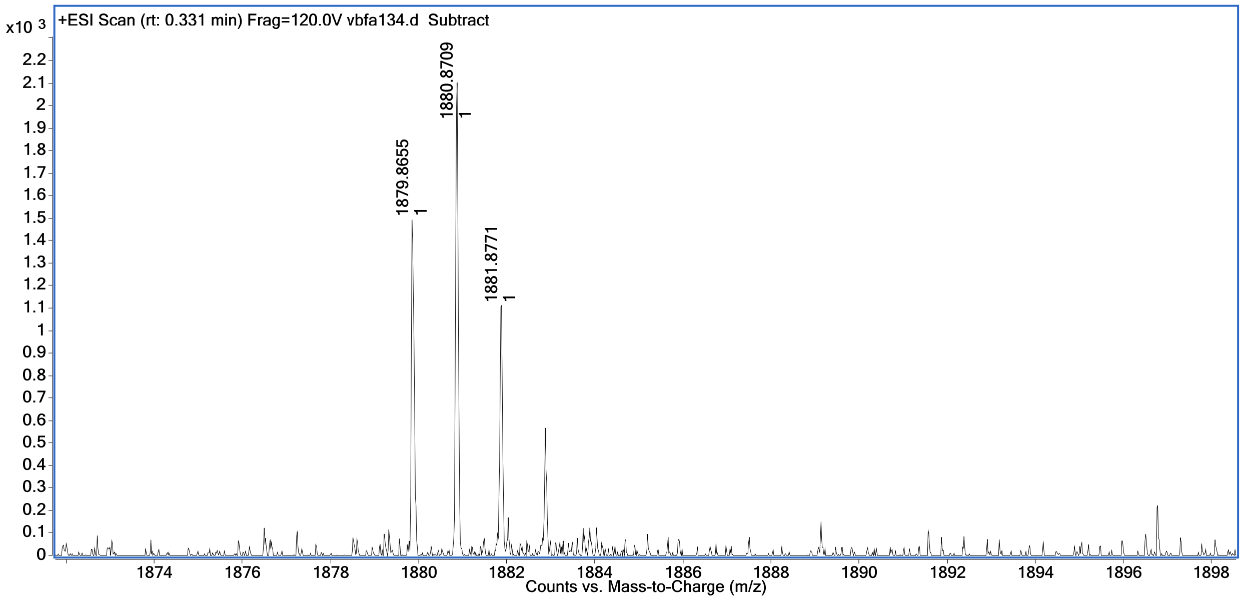
x10 3
+ESI Scan (rt: 0.331 min) Frag=120.0V vbfa134.d Subtract
1880.8709
1879.8655
1881.8771
Counts vs. Mass-to-Charge (m/z)

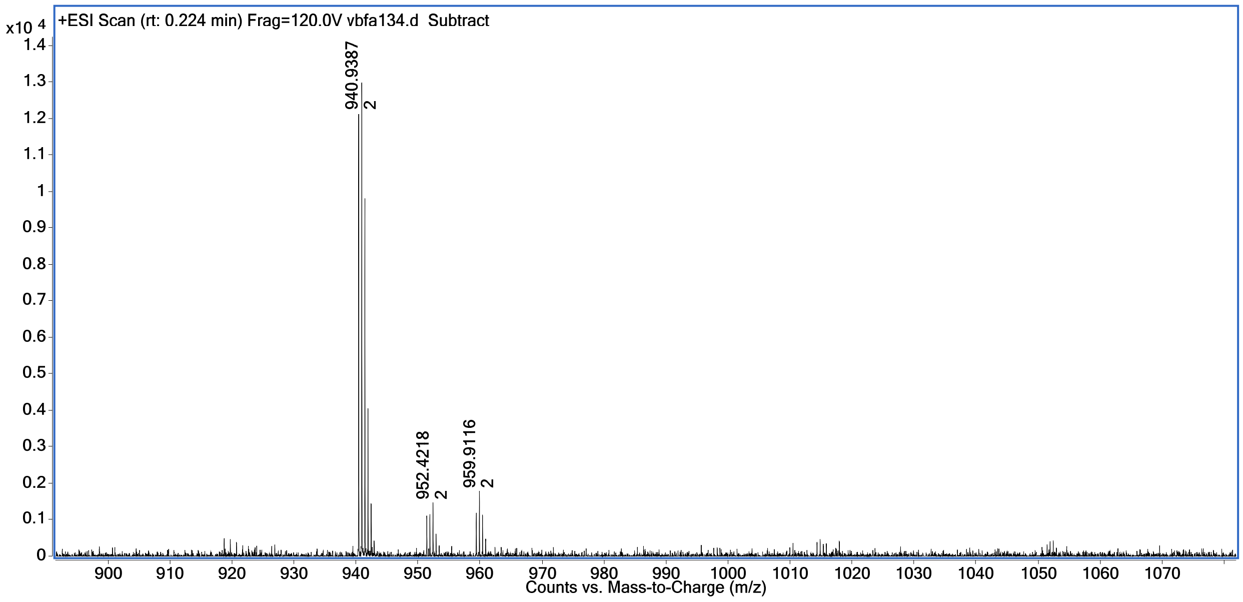

(e)

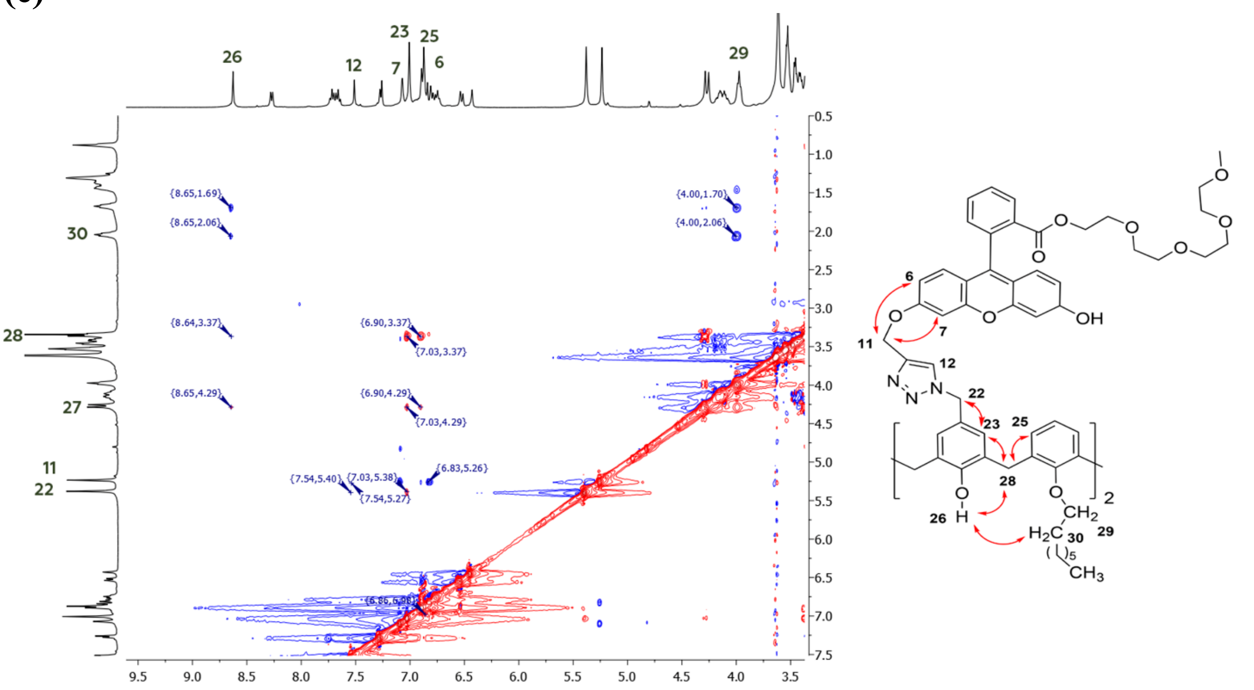

Figure S2. NMR ^{1}H (a), ^{13}C (b), FT IR (c), HRESI MS (d) and 2D NOESY NMR ^{1}H-^{1}H ($CDCl_3$) (e) spectra of *compound* ***(10)***.

(a)

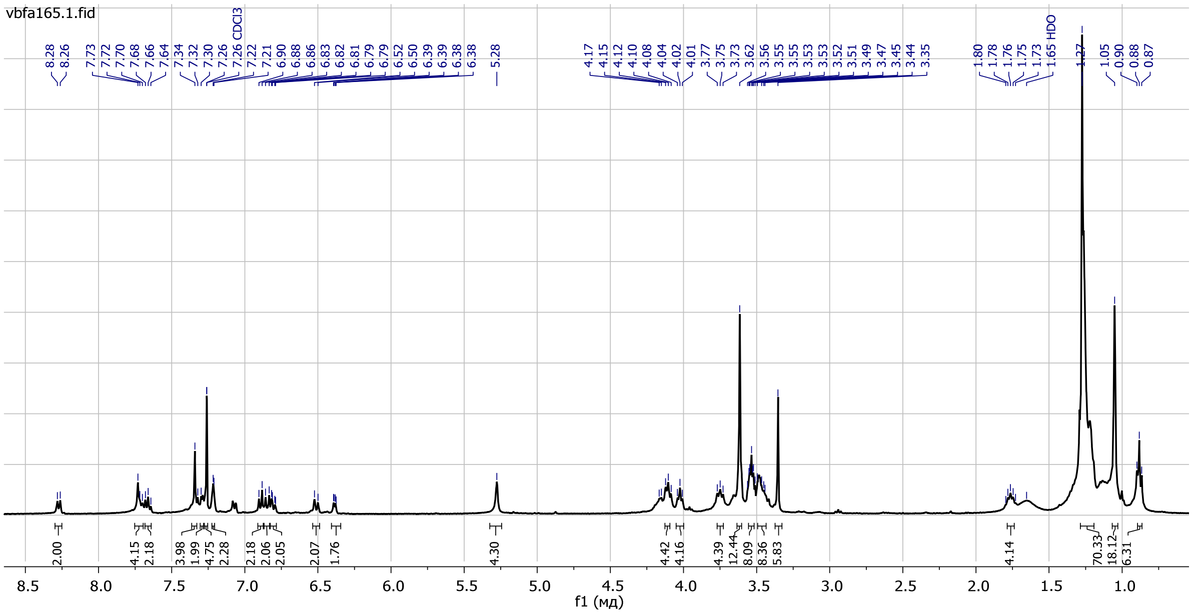
vbfa165.1.fid
8.28 8.26 7.73 7.72 7.70 7.68 7.66 7.64 7.34 7.32 7.30 7.26 CDCl3 7.22 7.21 6.90 6.88 6.86 6.83 6.82 6.81 6.79 6.79 6.52 6.50 6.39 6.39 6.38 6.38
5.28
4.17 4.15 4.12 4.10 4.08 4.04 4.02 4.01 3.77 3.75 3.73 3.62 3.56 3.55 3.55 3.53 3.53 3.52 3.51 3.49 3.47 3.45 3.44 3.35
1.80 1.78 1.76 1.75 1.73 1.65 HDO 1.27 1.05 0.90 0.88 0.87
2.00 4.15 2.18 3.98 1.99 4.75 2.28 2.18 2.06 2.05 2.07 1.76 4.30 4.42 4.16 4.39 12.44 8.09 8.36 5.83 4.14 70.33 18.12 6.31
8.5 8.0 7.5 7.0 6.5 6.0 5.5 5.0 4.5 4.0 3.5 3.0 2.5 2.0 1.5 1.0
f1 (мд)

(b)

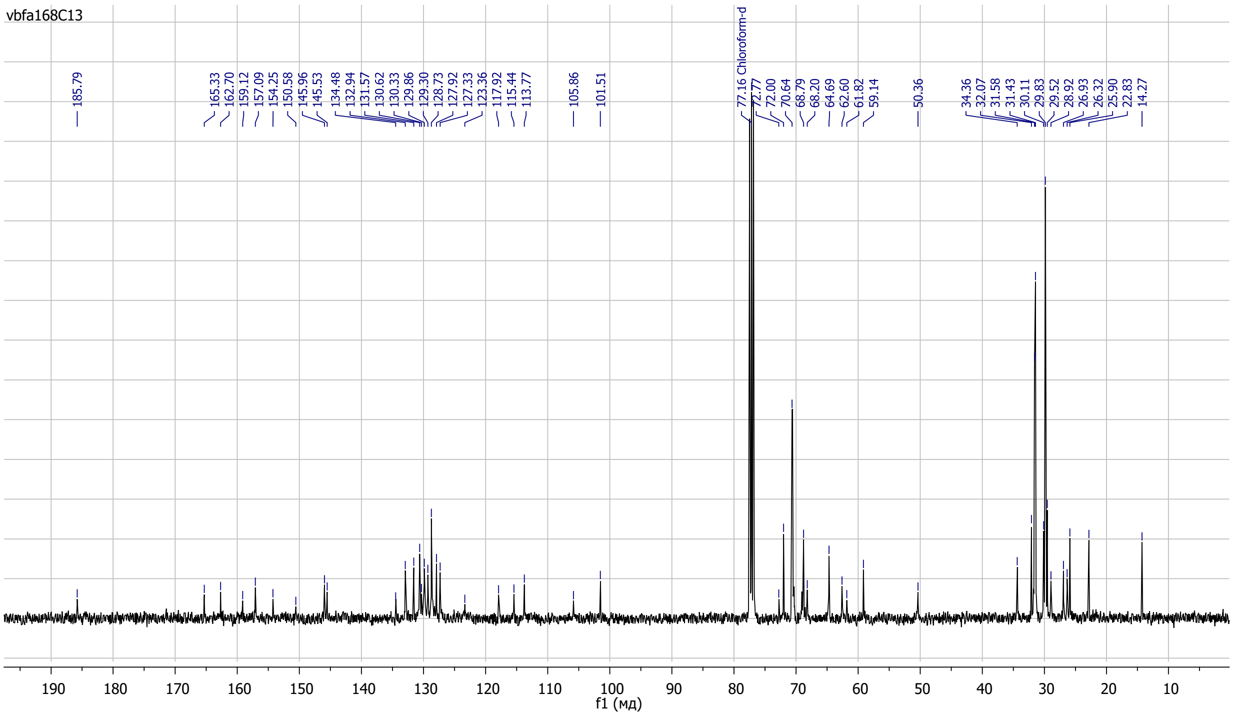
vbfa168C13
185.79
165.33 162.70 159.12 157.09 154.25 150.58 145.96 145.53 134.48 132.94 131.57 130.62 130.33 129.86 129.30 128.73 127.92 127.33 123.36 117.92 115.44 113.77
105.86 101.51
77.16 Chloroform-d 72.77 72.00 70.64 68.79 68.20 64.69 62.60 61.82 59.14
50.36
34.36 32.07 31.58 31.43 30.11 29.83 29.52 28.92 26.93 26.32 25.90 22.83 14.27
190 180 170 160 150 140 130 120 110 100 90 80 70 60 50 40 30 20 10
f1 (мд)

(c)

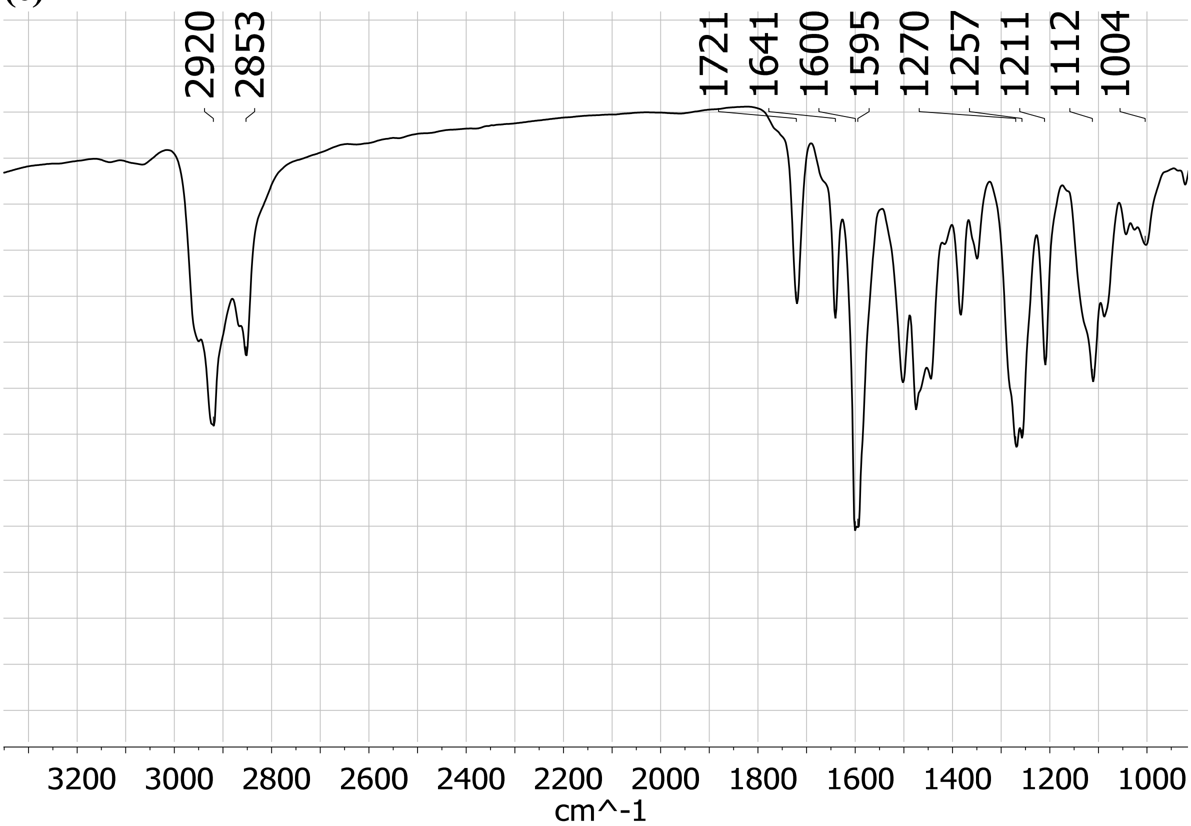
2920
2853
1721
1641
1600
1595
1270
1257
1211
1112
1004
3200
3000
2800
2600
2400
2200
2000
1800
1600
1400
1200
1000
cm^-1

(d)

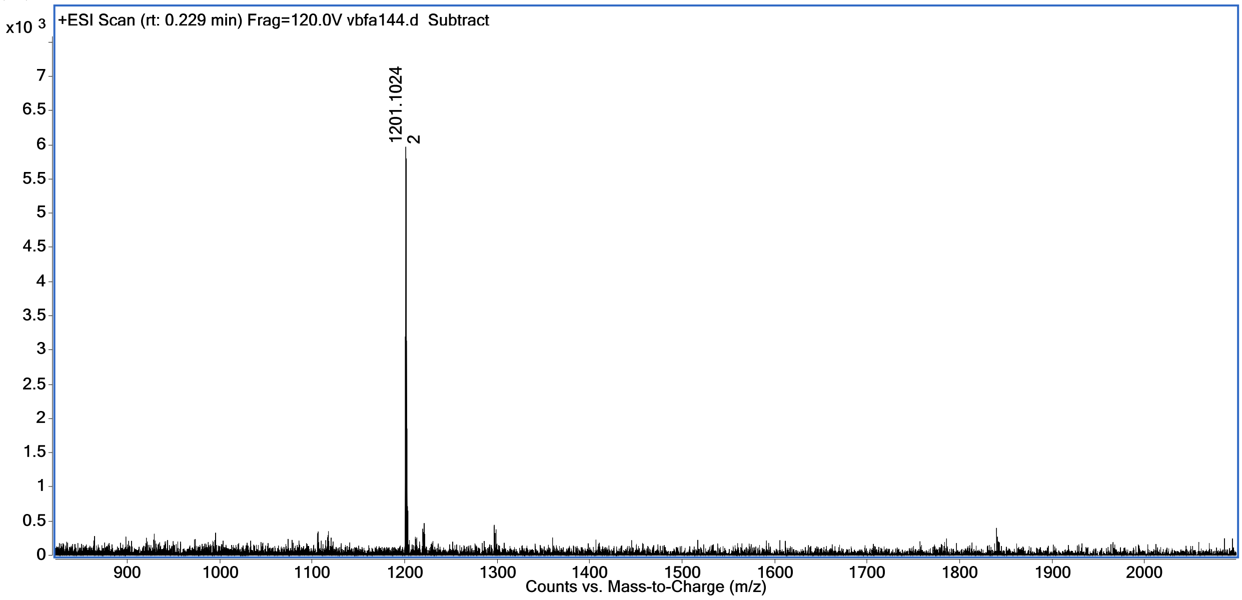
x10 3
+ESI Scan (rt: 0.229 min) Frag=120.0V vbfa144.d Subtract
1201.1024
2
Counts vs. Mass-to-Charge (m/z)

(e)

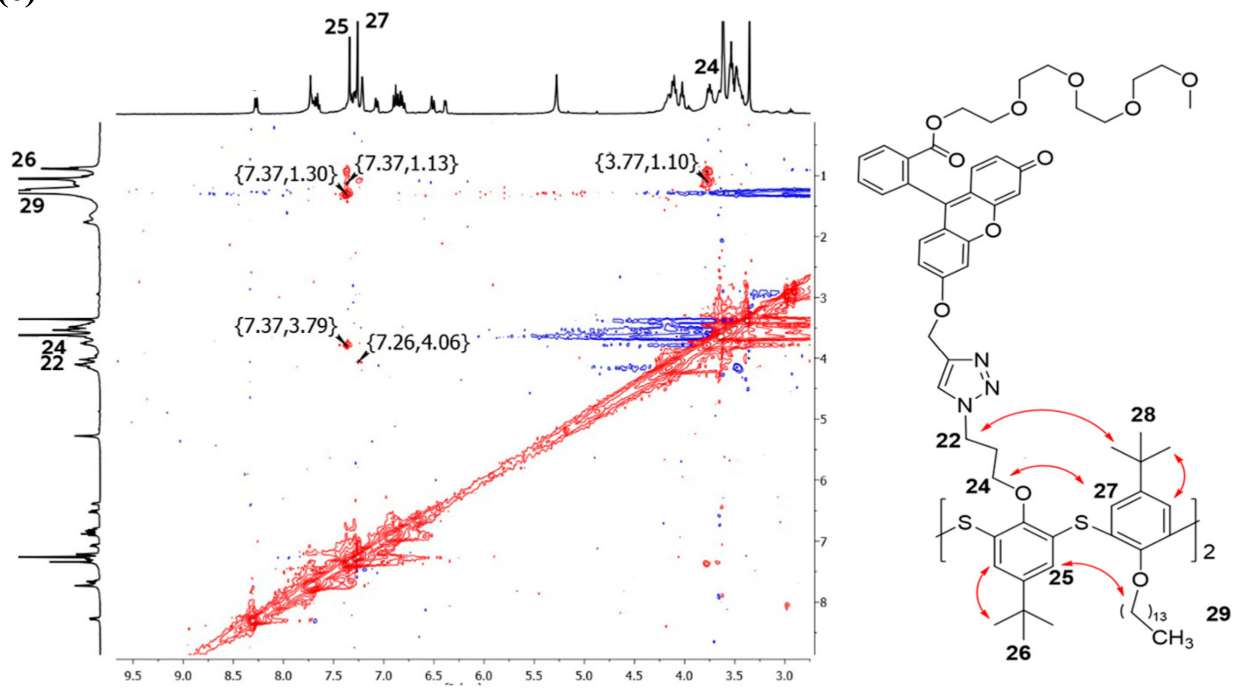

Figure S3. NMR ^{1}H (a), ^{13}C (b), FT IR (c), HRESI MS (d) and 2D NOESY NMR ^{1}H-^{1}H ($CDCl_3$) (e) spectra of *compound **(11)***.

(a)

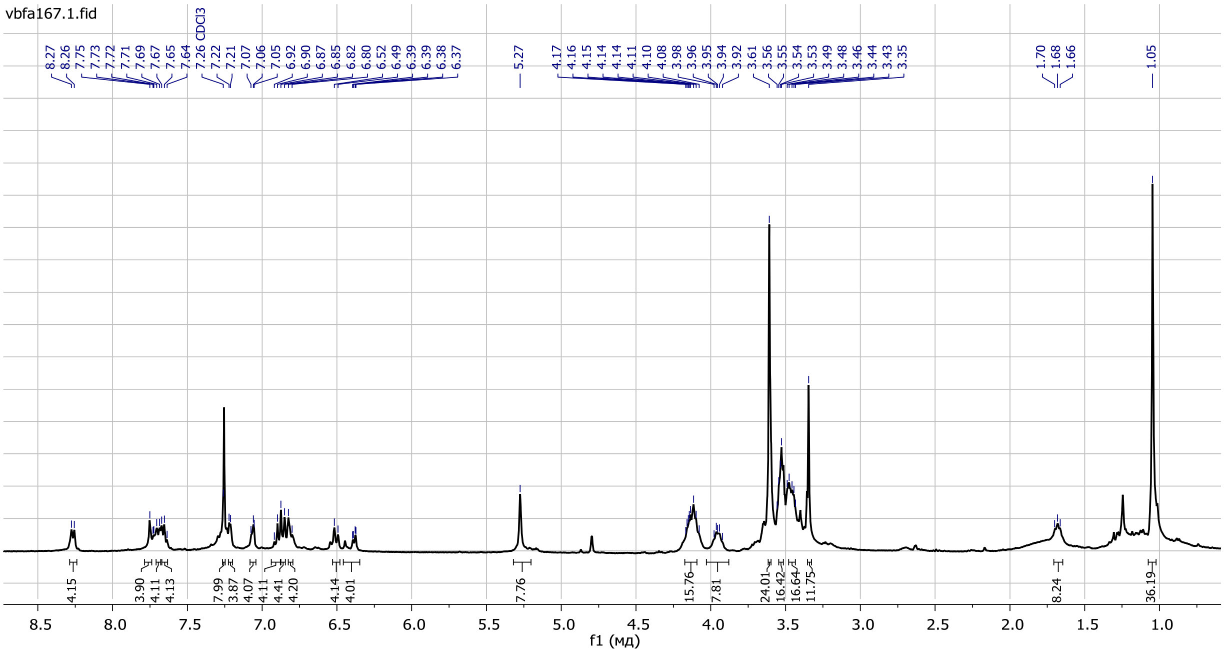
vbfa167.1.fid
8.27 8.26 7.75 7.73 7.72 7.71 7.69 7.67 7.65 7.64 7.26 CDCl3 7.22 7.21 7.07 7.06 7.05 6.92 6.90 6.87 6.85 6.82 6.80 6.52 6.49 6.39 6.39 6.38 6.37
5.27
4.17 4.16 4.15 4.14 4.11 4.10 4.08 3.98 3.96 3.95 3.94 3.92 3.61 3.56 3.55 3.54 3.53 3.49 3.48 3.46 3.44 3.43 3.35
1.70 1.68 1.66
1.05
f1 (мд)

(b)

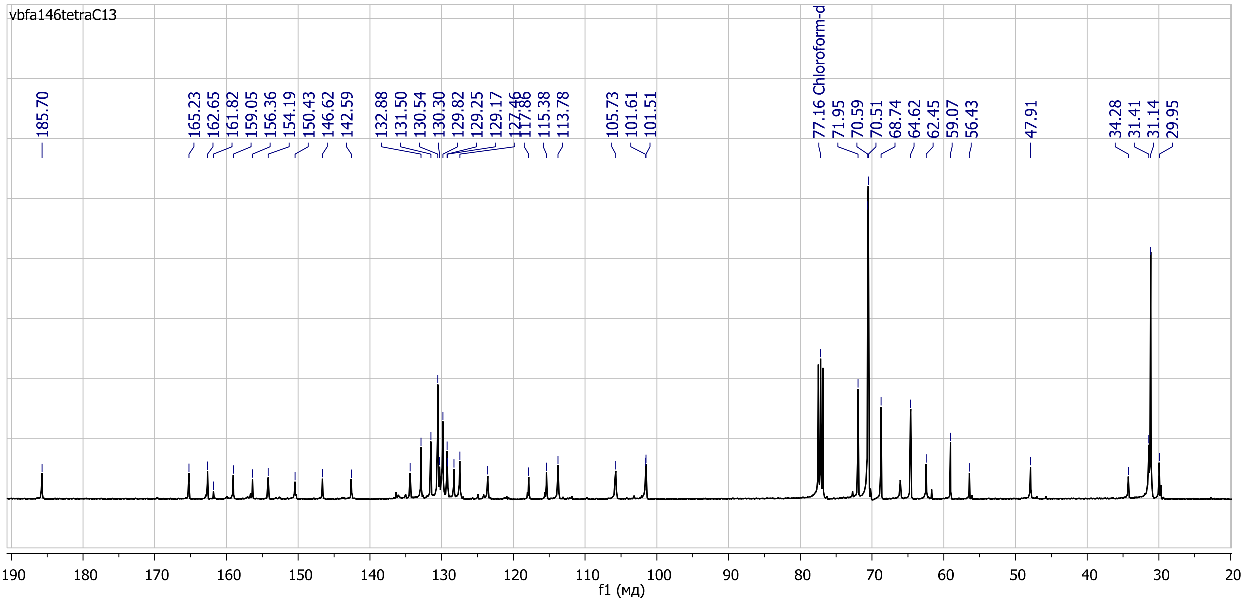
vbfa146tetraC13
185.70
165.23 162.65 161.82 159.05 156.36 154.19 150.43 146.62 142.59
132.88 131.50 130.54 130.30 129.82 129.25 129.17 127.46 117.86 115.38 113.78
105.73 101.61 101.51
77.16 Chloroform-d
71.95 70.59 70.51 68.74 64.62 62.45 59.07 56.43
47.91
34.28 31.41 31.14 29.95
f1 (мд)

(c)

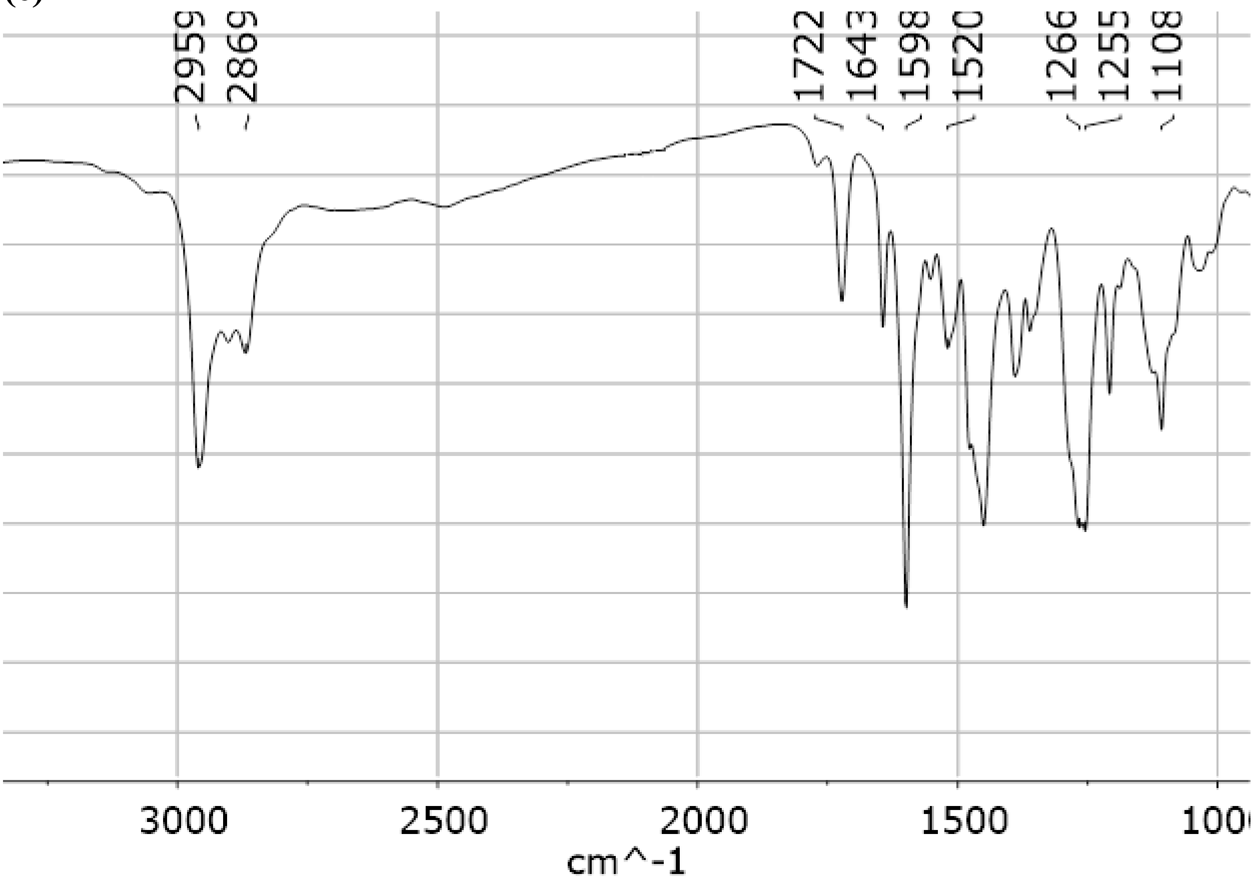

(d)

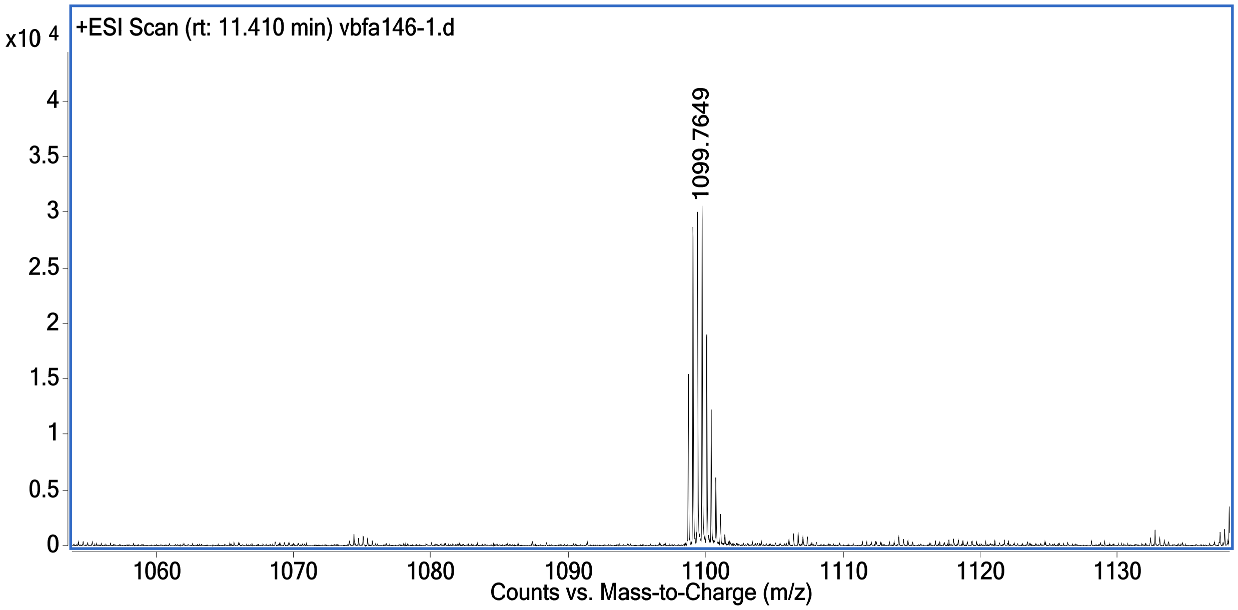

Figure S4. NMR ^{1}H (a), ^{13}C (b), FT IR (c) and HRESI MS (d) spectra of *compound (12)*.

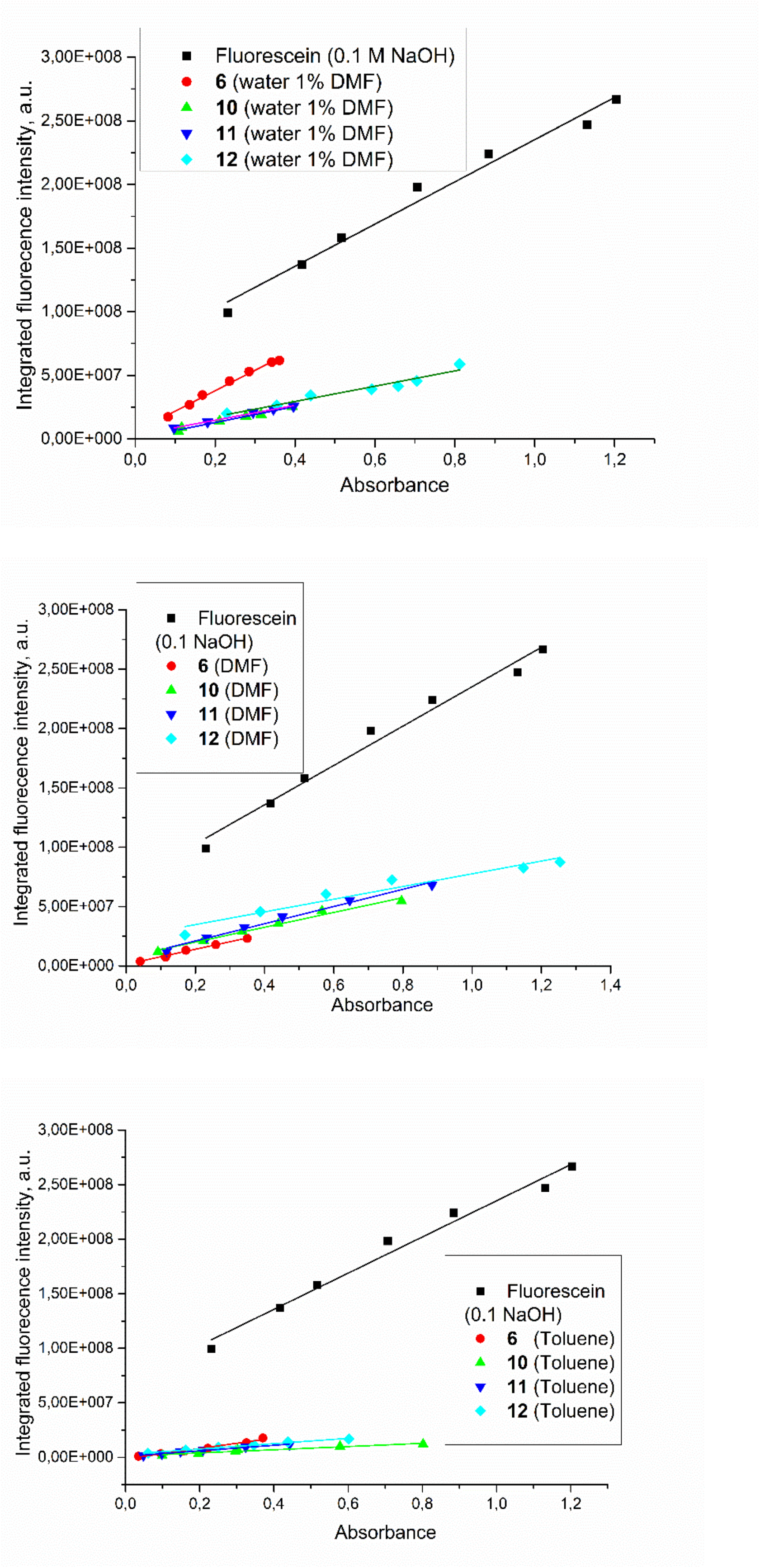

Figure S5. Integrated fluorescence intensity *vs* max. absorbance at different concentrations for fluorescein (0.1M NaOH) and compounds **6**, **10**, **11**, **12** (water with 1% DMF, pure DMF or toluene); C = 2.5-20 μM.

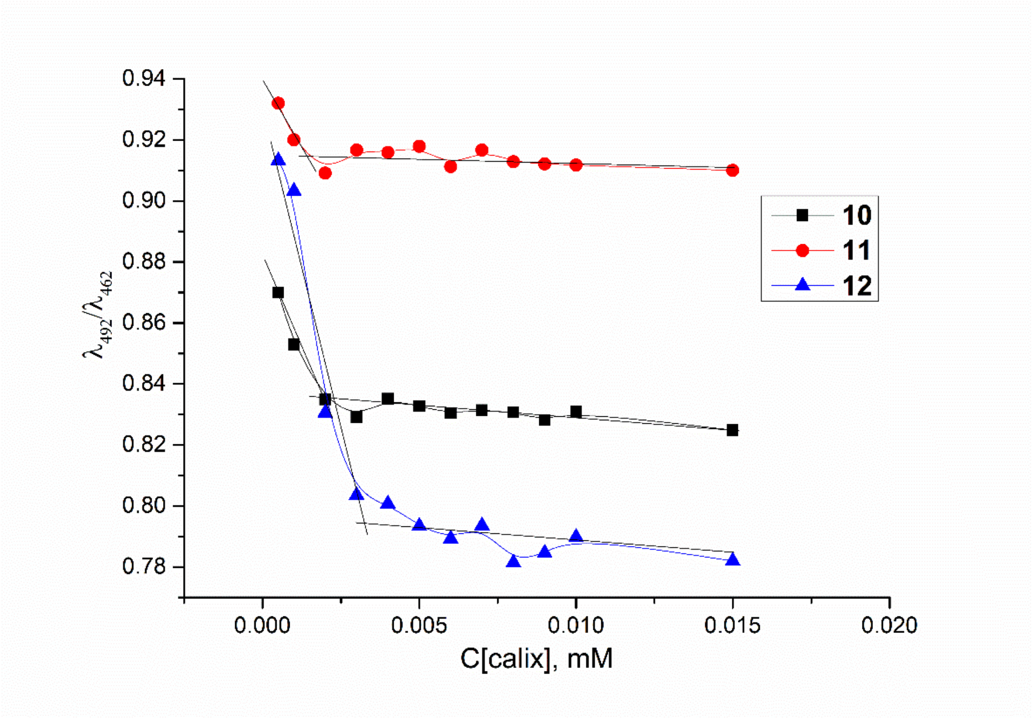

Figure S6. Ratio of absorption maxima at 492 and 462 nm *vs* calixarene **10**, **11**, **12** concentration (water with 1% DMF); C = 0.0005-0.015 mM.

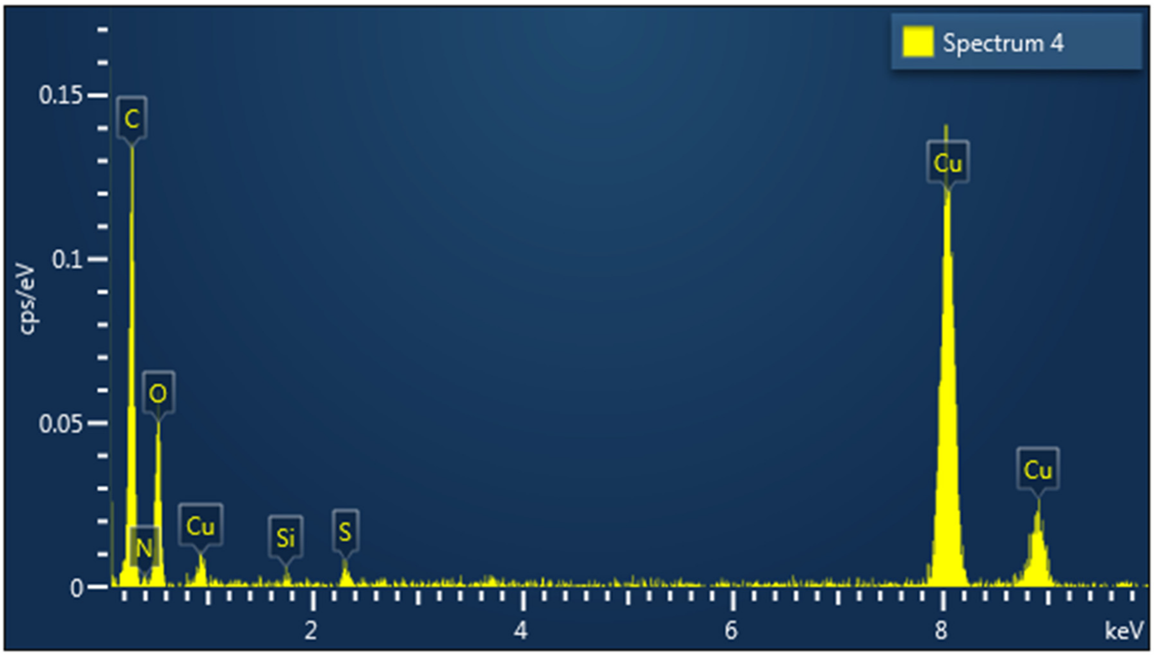

Figure S7. EDX spectrum of aggregates formed by **11**.

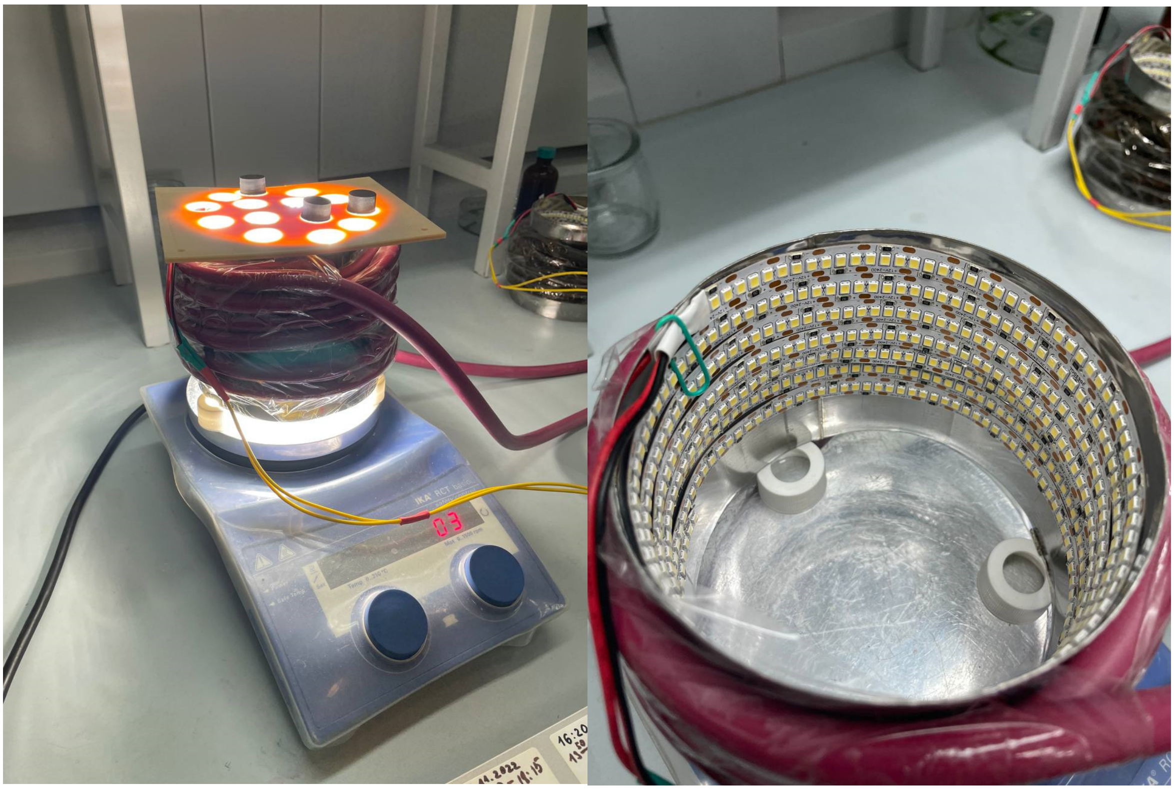

Figure S8. Photoreactor (white LED, 34 W)

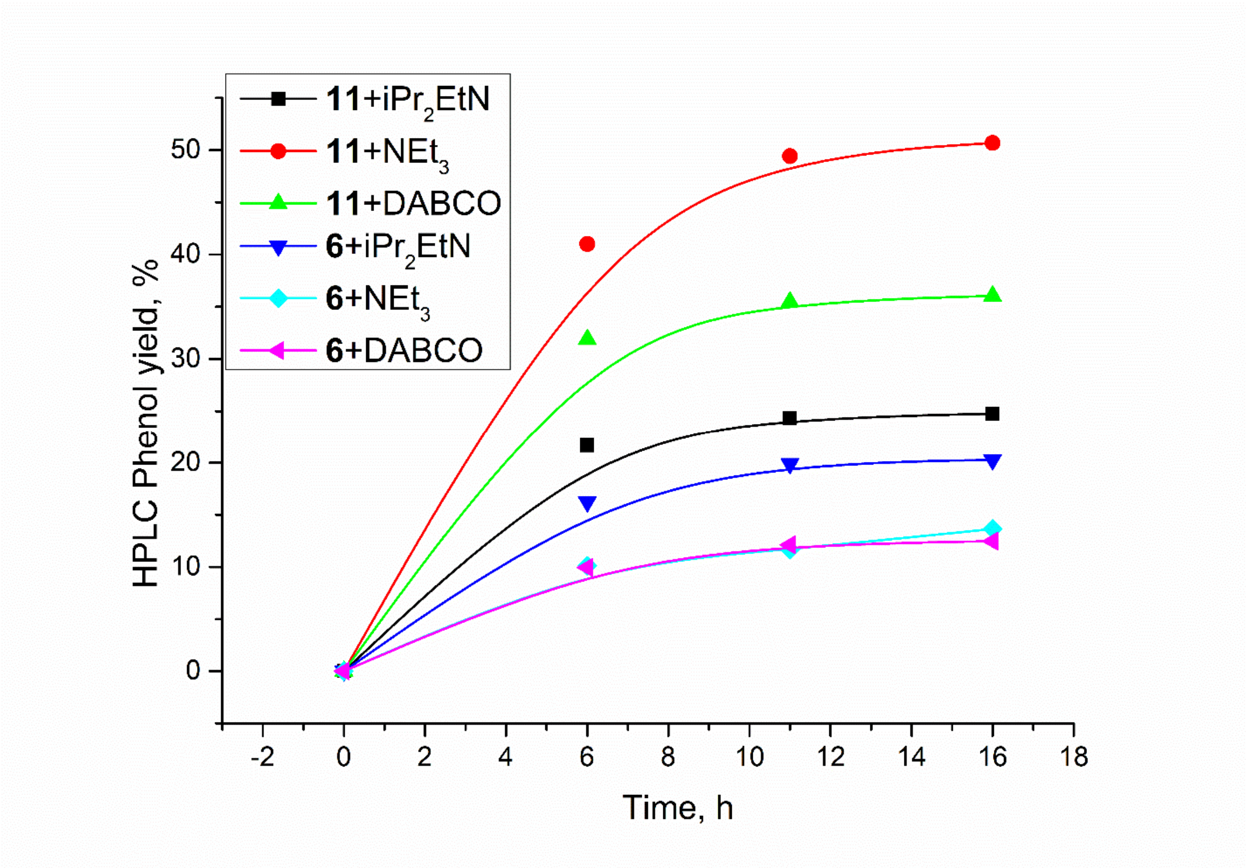

Figure S9. HPLC yield of phenol from photoredox ipso oxidative hydroxylation of phenylboronic acid using **6** and **11** *vs* time of irradiation (White LED, 34 W), 1%

DMF content with different bases C(PhB(OH$_2$) = 10 mM, C (base) = 20 mM, C (**6**, **11**) = 12 µM (A).

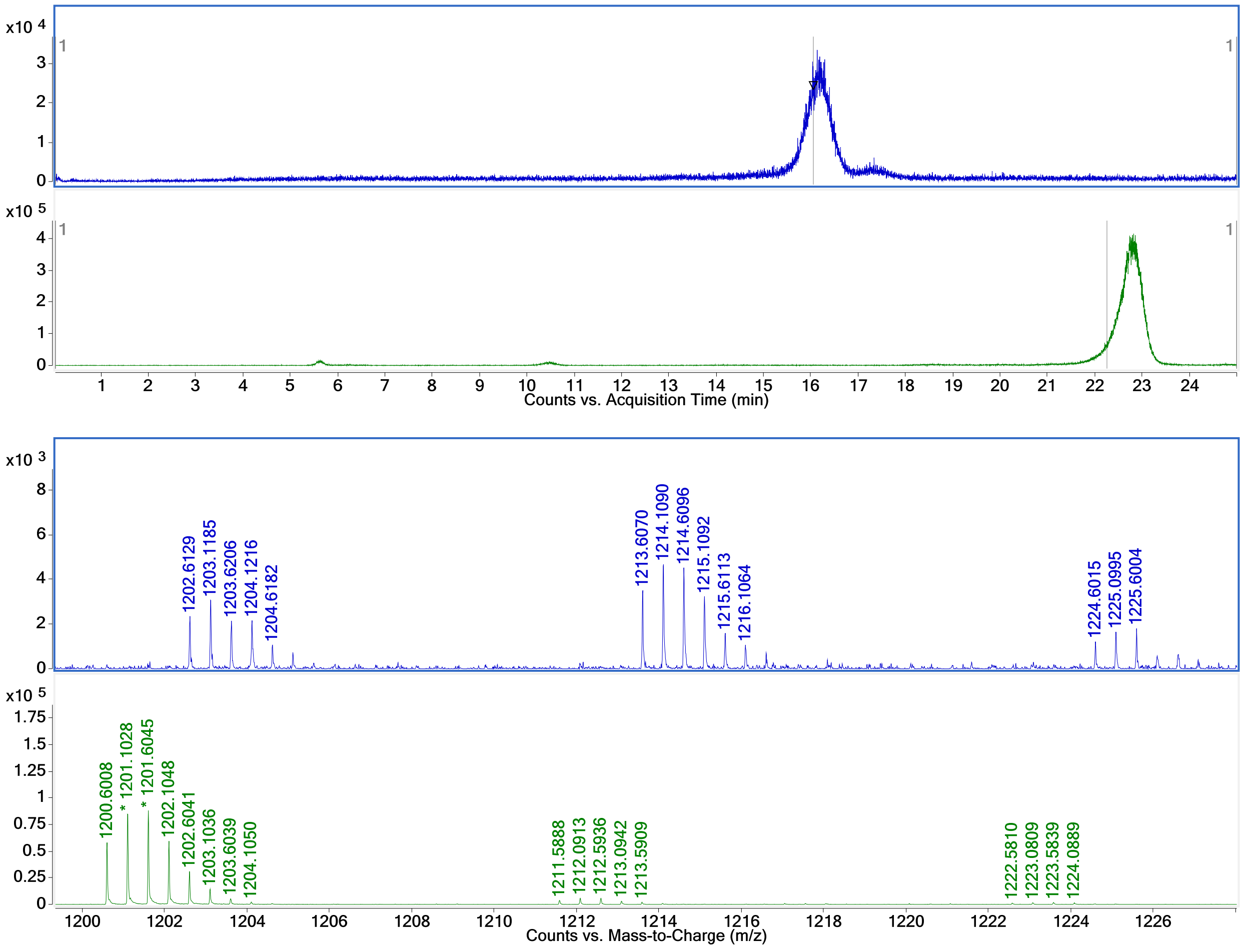

Figure S10. (Green) HPLC chromatogram and corresponding HRESI mass-spectra of **11** (0.0125 mM aqueous solution with 1% DMF) before (green) and after (blue) irradiation for 1 hour in the presence of NEt_3 (20 mM).
